# Evidence that the cold- and menthol-sensing functions of the human TRPM8 channel evolved separately

**DOI:** 10.1126/sciadv.adm9228

**Published:** 2024-06-21

**Authors:** Dustin D. Luu, Nikhil Ramesh, I. Can Kazan, Karan H. Shah, Gourab Lahiri, Miyeko D. Mana, S. Banu Ozkan, Wade D. Van Horn

**Affiliations:** ^1^ School of Molecular Sciences and The Virginia G. Piper Biodesign Center for Personalized Diagnostics, The Biodesign Institute, Arizona State University, Tempe, AZ, USA.; ^2^ Department of Physics and Center for Biological Physics, Arizona State University, Tempe, AZ, USA.; ^3^ School of Life Sciences, Arizona State University, Tempe, AZ, USA.

## Abstract

Transient receptor potential melastatin 8 (TRPM8) is a temperature- and menthol-sensitive ion channel that contributes to diverse physiological roles, including cold sensing and pain perception. Clinical trials targeting TRPM8 have faced repeated setbacks predominantly due to the knowledge gap in unraveling the molecular underpinnings governing polymodal activation. A better understanding of the molecular foundations between the TRPM8 activation modes may aid the development of mode-specific, thermal-neutral therapies. Ancestral sequence reconstruction was used to explore the origins of TRPM8 activation modes. By resurrecting key TRPM8 nodes along the human evolutionary trajectory, we gained valuable insights into the trafficking, stability, and function of these ancestral forms. Notably, this approach unveiled the differential emergence of cold and menthol sensitivity over evolutionary time, providing a fresh perspective on complex polymodal behavior. These studies provide a paradigm for understanding polymodal behavior in TRPM8 and other proteins with the potential to enhance our understanding of sensory receptor biology and pave the way for innovative therapeutic interventions.

## Teaser

Ancestral TRPM8 resurrection and cellular studies unveil keys to decoupling cold and menthol activation modes.

## INTRODUCTION

Two decades ago, two independent groups cloned and characterized transient receptor potential melastatin 8 (TRPM8) as a cold and menthol receptor and noted its implications in thermoregulation and pain (*1*, *2*). The discovery of TRPM8 explained the well-established correlation between menthol and cold sensitivity that had been characterized for at least 50 years prior (*3*). These two groups were recognized for these and other contributions with the 2021 Nobel Prize in Physiology or Medicine. While TRPM8 and the related hot-sensing capsaicin receptor transient receptor potential vanilloid 1 (TRPV1) have shown clinical efficacy for pain, their inhibition commonly leads to adverse thermal effects due to their coupled roles in temperature and pain perception. This feature of mechanistic coupling between temperature and ligand activation now limits their clinical application (*4*–*6*).

TRPM8 polymodal activation suggests that these two activation modes exhibit cross-talk (*7*–*10*), where menthol binding and cold temperature induce analogous mechanistic changes within the structure to gate (open) the channel. This begs the question of how both modalities emerged throughout the evolutionary history. Because of temperature changes throughout the earth’s history, we hypothesize that these two modalities emerged separately as TRPM8 polymodal behavior has evolved in certain lineages to meet specific environmental, sensory, and physiological requirements.

To identify and characterize the origins of human TRPM8 (hTRPM8) polymodal activation, we used ancestral sequence reconstruction (ASR) to evaluate key nodes along the hTRPM8 evolutionary trajectory. ASR has previously been used to study an acetylcholine receptor (AChR). AChR is a heteropentameric channel typically composed of two α subunits with β, δ, and ε subunits. The daCosta lab successfully resurrected an ancestral β subunit [~480 million years (Ma) ago] and characterized the hybrid extant/ancestral AChR channel with electrophysiology (*11*). A later study showed that the ancestral β subunit can form homopentamers (*12*), showcasing information that can be gained with ASR.

With extant hTRPM8 as a control, we resurrected the last common primate, mammalian, and vertebrate ancestors using ASR. These ancestral TRPM8 proteins were deliberately chosen. Previous phylogenetic analysis established that most thermosensitive TRP channels, including TRPM8, emerged in the most recent common vertebrate ancestor (*13*). Accordingly, we reconstructed the ancestral vertebrate TRPM8 (AncVert) sequence.

A key feature of mammalian physiology is endothermy (“warm-bloodedness”), which enables the internal generation of heat in response to the environment. In extant organisms, some endothermic underpinnings such as shivering and nonshivering thermogenesis rely on TRPM8 cold sensing (*1*, *2*, *14*). Comparative evolutionary physiology indicates that endothermy arose near the emergence of mammals (*15*). Accordingly, we reconstructed the sequence of ancestral mammalian TRPM8 (AncMam).

**Fig. 1.**
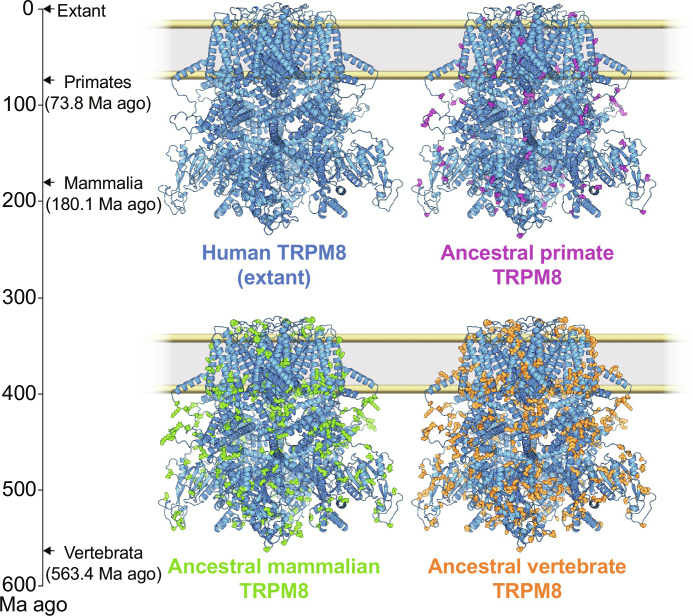
Ancestral TRPM8 reconstruction. The human evolutionary timeline from the emergence of vertebrates (**left**) was estimated from TimeTree5. AlphaFold2 models of full-length hTRPM8 (extant, blue). Residue changes from hTRPM8 identified in ASR shown for ancestral primate (AncPrim, purple), ancestral mammalian (AncMam, green), and ancestral vertebrate (AncVert, orange).

For the better studied TRPV1 hot and capsaicin receptor, differences between rodent and human proteins are well-documented (*16*, *17*). The less studied TRPM8 exhibits similar discrepancies between human and rodent proteins, affecting its function, regulation, and even response to small molecules (*18*–*20*). Considering the well-documented variations in TRPM8 function between rodents and humans, as well as the diverse thermal regulation mechanisms between these species (*21*–*24*), an ancestral node after the divergence of rodents should be informative. Therefore, we resurrected the ancestral primate TRPM8 (AncPrim). The estimated dates of the resurrected ancestral TRPM8 protein nodes and the molecular differences between extant hTRPM8 are shown in Fig. 1.

Resurrection and experimental analysis of these ancestral TRPM8 proteins validate that the orthologs are functional in heterologous mammalian cells. Flow cytometry–based plasma membrane trafficking studies and cellular thermal shift assays (CETSAs) show that the ancestral TRPM8 proteins retain similar trafficking and thermostability despite the number of molecular sequence differences between the extant hTRPM8 and the ancestral TRPM8 orthologs with evolutionary time. Whole-cell patch-clamp electrophysiology and calcium mobilization assays confirm our hypothesis that the cold and menthol responses differ in terms of emergence and sensitivity between the ancestral orthologs. Additional molecular insight from dynamic flexibility index (DFI) of extant and ancestral orthologs at room and cold temperatures provides further mechanistic insights into menthol and cold mode activation. These results show how ASR can be used as an alternative method to dissect polymodal protein behavior.

## RESULTS

### Sequences of ancestral TRPM8 orthologs are reconstructed

Allele-specific adaptive mutation candidates, including those associated with disease, can be derived from evolutionary information (*25*). Using a Bayesian-based phylogenetic approach, an evolutionary timetree was constructed from TRPM8 protein sequences derived from a diverse set of vertebrate species, mirroring previous phylogenetic analysis of the same species set (fig. S1) (*25*). The TRPM8-centric reconstruction generally reproduces canonical vertebrate evolution as assessed by comparing the branching patterns and species placement (*25*). Robust vertebrate phylogenetic tree construction determined exclusively from TRPM8 sequences gives confidence in the consensus TRPM8 ancestral node sequence quality. Accordingly, the protein sequences at key nodes along the human evolutionary trajectory were reconstructed for the ancestral TRPM8 primate, vertebrate, and mammalian nodes (fig. S2).

Reconstructed last common ancestral primate, mammalian, and vertebrate TRPM8 protein sequences differ from the extant hTRPM8 at 23–, 86–, and 124–amino acid positions per protomer, respectively (fig. S3). While the sequence variation is primarily well dispersed across TRPM8, the transmembrane domain (TMD; helices S1 to S6) is more conserved relative to the N- and C-terminal domains (tables S1 to S3). This result agrees with a previous analysis of exonic hTRPM8 single-nucleotide polymorphism frequency that showed the hTRPM8 TMD region is less tolerant to mutation (*26*). Several areas associated with polymodal TRPM8 function are conserved across the ancestral orthologs, including residues implicated in menthol binding (Y745, R842, and Y1005) (*27*–*31*), calcium binding (E782, Q785, Y793, N799, and D802) (*32*–*34*), and phosphatidylinositol 4,5-bisphosphate regulation (R851, N852, K995, R998, and R1008) (*35*–*38*).

The ancestral reconstruction also agrees with previously observed functional determinants of icilin activation. Icilin is a synthetic supercooling compound that elicits strong TRPM8 activation (*39*). The primary functional determinants of icilin sensitivity in hTRPM8 include N799, D802, and G805 in the S3 helix (*32*, *39*, *40*). AncVert has a G805A variant not found in AncMam, AncPrim, or extant hTRPM8. The G805A variant is commonly found in extant avian species that emerged after AncVert (~586 Ma ago) and before AncMam (~180 Ma ago) (*32*). As an example, chicken (*Gallus gallus*) TRPM8 icilin insensitivity can be rescued by an A796G, the equivalent residue position to 805 extant hTRPM8 (*39*). Similarly, frogs (*Xenopus laevis*), also emerged between AncVert and AncMam, are also icilin insensitive with an equivalent A841 (*41*). Nevertheless, because icilin is a modern artificial and synthetic compound, it is irrelevant to natural selection and, as such, was not explored in our studies. Together, these results give confidence in the ASR.

### Ancestral TRPM8 orthologs traffic and function in human cells

**Fig. 2.**
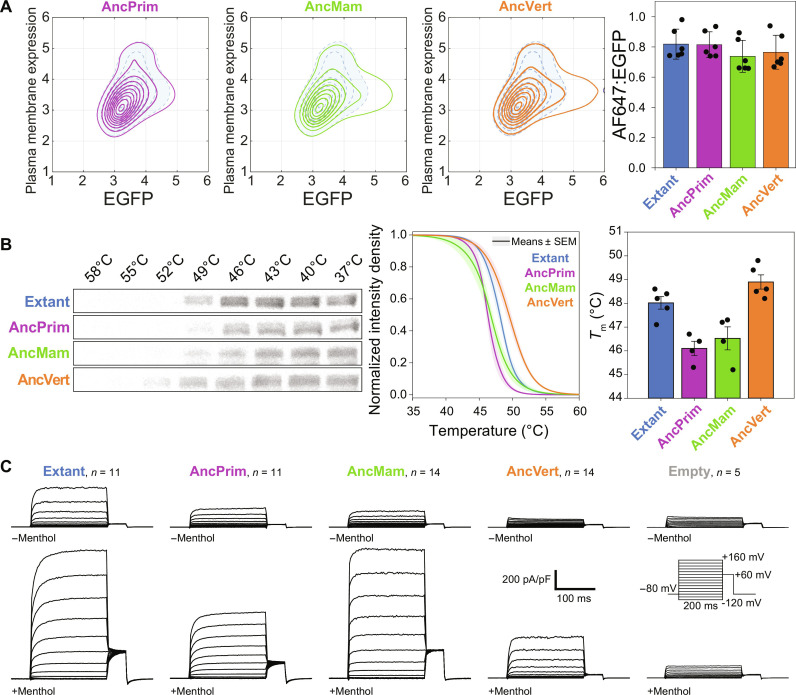
Resurrected ancestral TRPM8 orthologs traffic and function in human cells. (**A**) Resurrected ancestral TRPM8 orthologs traffic to the plasma membrane shows similar trafficking. Representative contours in logarithmic scale of flow cytometry data for the AncPrim (purple), AncMam (green), and AncVert (orange) against extant (blue). The number of cells used to make the contours was ungated and normalized to the lowest cell count of 12,607. The average ratio of Alexa Fluor 647 (AF647):enhanced green fluorescent protein (EGFP) are from EGFP^+^ gated cells with ratios and SD of 0.82 ± 0.10, 0.81 ± 0.9, 0.74 ± 0.11, and 0.76 ± 0.11 for extant, AncPrim, AncMam, and AncVert, respectively. The averages are from six biological replicates conducted on different days, and individual averages of each replicate are jittered. (**B**) Thermal stability of ancestral TRPM8 orthologs. Representative CETSA Western blots using anti-HA antibody and enhanced chemical luminescence. Combined stability curves show minor differences in stability between the ancestral TRPM8 orthologs with a modest 2°C shift in melting temperature (*T*_m_) when compared to extant (48.0 ± 0.3°C) and AncVert (48.9 ± 0.3°C) with AncPrim (46.1 ± 0.3°C) and AncMam (46.5 ± 0.5°C). Four or five biological sets of CETSA-independent experiments were carried out, with experiments conducted on different days. Errors are in SEM. (**C**) Patch-clamp electrophysiology of TRPM8 in the absence (top) and presence (bottom) of the cognate TRPM8 agonist menthol (500 μM) indicates that it is a functional ion channel. These trafficking, stability, and functional studies were conducted in HEK-293 cells. Statistical analyses are listed in table S4.

Canonical hTRPM8 resides in the plasma membrane, where it functions in various physiological and pathophysiogical roles (*14*, *42*, *43*). To ensure rigorous functional characterization of the resurrected ancestral TRPM8 orthologs, we performed flow cytometry–based immunostaining studies to quantify trafficking and validate that the resurrected orthologs are compatible with extant human cellular membrane protein biogenesis, quality control, and plasma membrane trafficking pathways (*44*–*46*). Trafficking studies show similar trafficking for the extant and ancestral TRPM8 orthologs under equivalent conditions (Fig. 2A and fig. S4). The trafficking differences of extant, AncPrim, AncMam, and AncVert TRPM8 orthologs are not statistically significant using an unpaired *t* test.

We also performed CETSAs to gauge the thermal stability of the protein. Traditionally, CETSA is used in drug discovery to evaluate drug target engagement in cells and tissues (*47*–*49*). In this context, we evaluate the relative stability of extant and ancestral hTRPM8 proteins sans ligands to quantify their relative stabilities in human embryonic kidney 293 (HEK-293) cells. CETSA analysis shows minor differences in thermal stability (Fig. 2B and fig. S5). While we interpret CETSA data as a proxy for stability, we note that there are caveats in this interpretation. These can arise from complexities in trafficking and proteostasis. In addition, the observed protein thermal stability can be complicated by molecular events such as if unfolding does not lead to protein aggregation or antibody recognition variability (*49*). Nevertheless, the subtle changes in stability are remarkable, especially given the substantial sequence variance between the extant and ancestral TRPM8 orthologs. Accordingly, these data show that the trafficking and thermal stability between these proteins remain similar across evolutionary time.

To evaluate the functionality of extant and ancestral TRPM8 orthologs, whole-cell patch-clamp electrophysiology was performed in the absence and presence of menthol, the cognate hTRPM8 chemical agonist (Fig. 2C and fig. S6) (*1*, *2*). In the absence of agonist stimulus, the voltage-stimulated TRPM8 current density is diminished backward in time. Menthol-stimulated current densities vary in a more complicated pattern, with AncMam showing the fastest activation kinetics, most robust current densities, and largest fold increase in menthol activation, while AncVert, the most ancient, is the least menthol sensitive. Nevertheless, all ancestral TRPM8 orthologs show substantial menthol activation relative to a negative control (Fig. 2C and fig. S6). Despite the many mutations relative to extant hTRPM8, the resurrected ancestral TRPM8 orthologs are trafficking-compatible, are stable, and function in human cells. This sets the stage for comparative studies of relative cold and menthol activation along the hTRPM8 evolutionary trajectory.

### Temperature-dependent studies of ancestral TRPM8 identify the emergence of cold activation

**Fig. 3.**
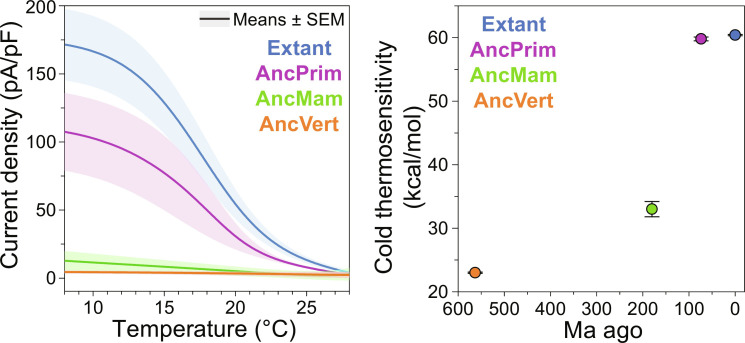
Thermosensitivity of ancestral TRPM8 orthologs. Temperature-dependent whole-cell patch-clamp electrophysiology measurements show step-wise decreases in thermosensitivity for ancient TRPM8 orthologs (**left**). Current density was recorded from temperature ramps from 28° to 8°C at 60 mV and subtracted against the empty pIRES2 vector to remove any endogenous temperature effects (fig. S7A). The data are the averages of 8, 9, 11, and 8 biological replicates for extant (blue), AncPrim (purple), AncMam (green), and AncVert (orange), respectively. Quantification of the cold thermosensitivity (|Δ*H*|) with SE as a function of evolutionary time indicates that TRPM8 cold sensitivity emerged later in the human evolutionary trajectory (**right**). Statistical analyses are listed in table S4.

Extant hTRPM8 functions as a molecular thermometer that is exquisitely sensitive to and gated by cold temperatures (*1*, *2*). Temperature-dependent whole-cell patch-clamp electrophysiology was used to evaluate resurrected ancestral hTRPM8 protein cold sensitivity (Fig. 3 and fig. S7). Cold-temperature ramps from 28° to 8°C produce robust current densities in extant hTRPM8. The inherent cold activation of ancestral TRPM8 orthologs decreases stepwise, with AncVert being the least cold-sensitive ortholog.

TRP channel thermosensitivity is commonly quantified by measuring the change in pseudo standard state enthalpy (Δ*H*) between the closed and temperature-induced open states, with negative Δ*H* values expected for cold sensors (*50*–*53*). The cold thermosensitivity (Δ*H*) for the extant, AncPrim, AncMam, and AncVert are −60.4 ± 0.1, −59.8 ± 0.3, −33.0 ± 1.2, and −23.0 ± 0.1 kcal/mol, respectively. Evaluation of the extant human and ancestral TRPM8 cold sensitivity as a function of evolutionary time shows a substantial jump that emerged between the AncMam (~180 Ma ago) and AncPrim (~74 Ma ago) as shown in Fig. 3. Given that extant TRPM8 orthologs have been shown to correlate to environmental thermal niches (*41*, *54*) and another ASR study identified relationships between proteins and ancient earth temperatures (*55*), we evaluated the ancestral TRPM8 thermosensitivity in the context of historical earth temperatures (fig. S8) (*56*–*58*). These analyses show no clear correlation between the emergence of TRPM8 cold activation and the estimated earth temperatures, indicating that earth temperature was not likely a major driver of cold sensitivity.

Some extant TRP channels have shown species-dependent divergent thermoactivation. For example, human transient receptor potential channel subfamily A member 1 (TRPA1) functions as a hot sensor (*59*), while the rodent orthologs are cold-activated (*60*). TRPA1 thermosensitivity can be swapped with only a few discreet mutations, as evidenced by a mouse TRPA1 study that changed the channel from cold to heat sensing (*61*). Given the TRPA1 thermosensing promiscuity and the low cold sensitivity of AncVert TRPM8, we evaluated extant human and ancestral TRPM8 proteins as putative warm/hot sensors with electrophysiology measurements stepped from 25° to 40°C (fig. S9). Negative control experiments show increased current density with the elevated temperature. In contrast, all TRPM8 orthologs show the opposite trend confirming that each ortholog is cold-activated and that none are promiscuous hot sensors under normal biologically relevant temperatures. Extant hTRPM8 shows the most marked decrease in current density with increased temperature stimulation with a ~5.4-fold decrease compared to less than 2-fold decrease in the ancestral TRPM8 orthologs (fig. S9).

Given the substantial jump in thermosensitivity between AncMam and AncPrim TRPM8, we evaluated the residue differences between these orthologs. This analysis indicates that the 62 residue variants that endow AncPrim cold sensitivity are widely dispersed across the protein (fig. S10). Between AncMam and AncPrim, we note that there are two mutations, which, in previous studies, have markedly altered cold activation in extant TRPM8 orthologs, specifically V915Y and R897E (*62*, *63*). Emperor penguin (*Aptenodytes forsteri*) TRPM8 has a Y915 equivalent residue (Y919 in penguins) and exhibits lower cold-stimulated currents than African elephant (*Loxodonta africana*) TRPM8 with a V915 (V919 in elephants) equivalent (*62*). Introducing the equivalent opposing mutations in penguin (Y919V) and elephant (V919Y) TRPM8 reversed the respective current densities, with penguin and elephant TRPM8 increasing and decreasing cold sensitivity, respectively. Similarly, chicken TRPM8 (residue E897 in rat/human and E887 in chicken) exhibits diminished cold sensitivity relative to the mouse equivalent (R897), and swapping the chicken residue into mouse TRPM8 (R897E) decreased cold activation (*63*). The difference in cold sensitivity between AncPrim and AncMam could be partly explained by the V915Y and R897E mutations observed in other nonhuman extant species and will require future studies. Nevertheless, our data indicate that cold activation was an emergent property that arose between mammalian and primate evolution.

Previous studies of extant TRPM8 orthologs identified that side-chain hydrophobicity of the pore domain (PD) correlated with environmental habitat temperature and reflected TRPM8 cold sensitivity (*62*). We evaluated the change in side-chain hydrophobicity of the ancestral TRPM8 orthologs relative to extant hTRPM8 with three distinct hydrophobicity scales (fig. S11 and tables S5 and S6) (*64*–*66*). Analysis of the TRPM8 membrane domains, including the S1-S4 voltage-sensing–like domain (VSLD), the S5-S6-TRP helix PD, and the combined VSLD and PD TMD, shows that the PD of ancestral TRPM8 proteins becomes more hydrophobic with increased cold sensitivity, following the trend identified in various extant TRPM8 orthologs (*62*). The VSLD, which has also been implicated in TRPM8 cold sensing (*29*) and TRPV1 hot sensing (*50*), generally shows the opposite trend of the PD where the VSLD becomes less hydrophobic relative to extant hTRPM8 with increasing cold activation. The PD and VSLD show opposite trends, effectively compensating for the changes in hydrophobicity. The changes in hydrophobicity have no clear relationships with menthol sensitivity (fig. S11). We interpret this to indicate that the inherent drivers of cold and menthol activation are separate from each other and should be separable. We also evaluated the biological hydrophobicity free energy transfer of the full-length and various domains of these TRPM8 orthologs against the CETSA-derived melting temperatures (*T*_m_) values (table S6). Our goal was to gain insights into translocon-mediated membrane integration. We observed no clear correlations between the transfer free energy and the *T*_m_ values.

### TRPM8 menthol sensitivity emergence is distinct from that of cold

**Fig. 4.**
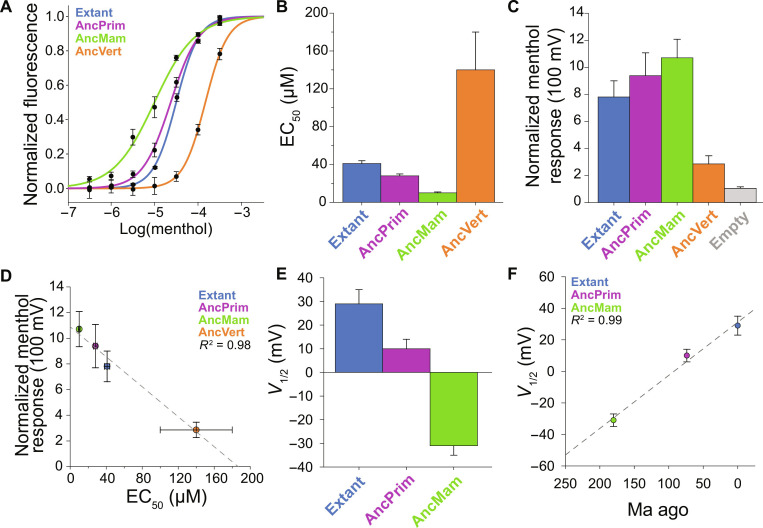
Ancestral TRPM8 orthologs are menthol receptors with variable menthol affinity. (**A**) Menthol response curves with SEM of the ancestral TRPM8 orthologs from calcium mobilization assay with addition of menthol. (**B**) The menthol half-maximal effective concentration (EC_50_) (potency) was calculated from Fluoforte-based Ca^2+^ flux data of nine biological replicates with the values plotted with SE. All EC_50_ values had a *P* value of <0.0001 with each other using an unpaired *t* test. (**C**) The relative menthol efficacy (*E*) with SEM of ancestral TRPM8 orthologs was estimated from current-density fold increase from patch-clamp measurements in the absence and presence of 500 μM menthol. (**D**) *E* varies linearly (*R*^2^ = 0.98) with EC_50_ indicative of variable affinity (*K*_d_), where AncMam (green) has the highest relative affinity, potency, and efficacy for menthol. (**E**) The *V*_1/2_ with SE of the voltage-menthol coupling obtained from the whole-cell patch-clamp tail currents. (**F**) *V*_1/2_ shows a linear relationship (*R*^2^ = 0.99) with the emergence of extant, AncPrim, and AncMam TRPM8. Whole-cell patch-clamp electrophysiology data are from 8 to 11 biological replicates collected over multiple days and transfections. Statistical analyses are listed in table S4.

TRPM8 is the cognate receptor for the monoterpene cooling compound menthol. Figure 2C and fig. S5 show that the resurrected nodes along the hTRPM8 evolutionary trajectory exhibit variable menthol sensitivity. In particular, the dependence of menthol-stimulated electrophysiology current density follows a distinct trend from cold activation, where AncMam TRPM8 is most sensitive to a saturating concentration of menthol (500 μM) for extant hTRPM8. To validate this result and further investigate menthol activation of TRPM8 orthologs, we performed fluorescence-detected calcium flux assays in HEK-293 cells to determine the half-maximal effective concentration (EC_50_) of menthol for each resurrected node. The menthol response curves and resulting EC_50_ are shown in Fig. 4 (A and B) and fig. S12. AncMam TRPM8 has the lowest EC_50_ (highest potency) of 10 ± 1 μM among those tested, with the more ancient AncVert having the weakest menthol potency of 140 ± 40 μM. Further analysis of the whole-cell patch-clamp electrophysiology menthol responses (Fig. 4C) highlighting the ratio of current densities in the absence and presence of menthol shows a related trend, with the largest response from AncMam TRPM8 with a 10.7 ± 1.4–fold increase and the smallest relative response from AncVert TRPM8 with only a 2.9 ± 0.6–fold increase. The electrophysiology data highlight differences in menthol efficacy (*E*), whereas the EC_50_ data reflect a distinct aspect of the pharmacology, the potency. In a simple del Castillo–Katz model, *E* and EC_50_ are related by the affinity of the receptor to the ligand [equilibrium dissociation constant (*K*_d_)], where *K*_d_ = EC_50_(1 + *E*) (*31*, *67*). A plot of the EC_50_ versus *E* TRPM8 values shows a linear relationship [Fig. 4D, coefficient of determination (*R*^2^) = 0.98], indicating that the difference in menthol activation arises from distinct affinity (*K*_d_) for the cognate agonist. As mentioned above, the canonical binding site residues were conserved from AncVert to extant hTPRM8. This indicates that menthol affinity is regulated allosterically from the orthosteric binding site. We note that this is a feature seen in other proteins (*68*–*76*). These studies indicate that menthol ligand sensitivity along the human evolutionary trajectory peaked near AncMam TRPM8 (~180 Ma ago), whereas cold activation reached a near steady state with AncPrim TRPM8 (~75 Ma ago).

TRPM8 is weakly voltage sensitive, where voltage is a partial channel activator of the channel (*77*). In extant mammalian TRPM8 channels, menthol has been shown to shift voltage activation toward physiological resting membrane potentials (*10*). We sought to test whether increased voltage coupling could contribute to AncMam menthol sensitivity. Steady-state current of TRPM8 orthologs in the absence of menthol shows diminishing currents correlating with time from present (Fig. 2C). Extant hTRPM8 tail current measurements in the presence of 500 μM menthol identify the midpoint of voltage activation (*V*_1/2_) of 29 ± 6 mV, relatively far from the resting potentials of human cells (Fig. 4E and fig. S13). AncPrim and AncMam have substantial leftward shifts toward more negative potentials with Δ*V*_1/2_ values of −19 ± 4 and −60 ± 4 mV relative to extant hTRPM8, respectively. The *V*_1/2_ values for AncMam, AncPrim, and extant hTRPM8 correlate extremely well with evolutionary time (Fig. 4F, *R*^2^ = 0.99), suggesting that voltage-menthol coupling was selected against during hTRPM8 evolution. We note that a loss of TRPM8 voltage coupling–based activation would likely be required to enable the high cold sensitivity of hTRPM8, a feature that has been hypothesized previously (*53*).

Previous studies of hTRPM8 identified that mutating positively charged residues in the S4 transmembrane helix and the S4-S5 linker (R842A, H845A, R851Q, R862A, and K856R) generally shifted the *V*_1/2_ to more positive potentials at 25°C (*29*). It is noteworthy, however, that extant human residues are conserved across the ancestral TRPM8 orthologs. Consequently, these mutations do not explain the shift in *V*_1/2_ seen in the ancestral TRPM8 orthologs. While future studies are required to ascertain the underpinnings of voltage-menthol coupling, the correlated electrophysiology and calcium mobilization assay data indicate that the menthol sensitivity differences arise from differences in binding affinity and altered coupling with voltage activation.

### DFI provides molecular insights

Given the differential menthol and cold activation profiles along the hTRPM8 evolutionary trajectory (Figs. 2 to 4), we used molecular dynamics (MD) simulations of the TRPM8 evolutionary orthologs to provide additional molecular insight. In particular, the DFI was calculated from MD trajectories to quantify the per-residue flexibility profile of the TMD (*69*, *75*, *78*–*81*). DFI combines MD simulation with force perturbations from perturbation response scanning (PRS) and linear response theory (LRT) to evaluate each residues response to a force perturbation at all other locations in the protein (*69*, *71*, *74*, *79*–*85*). Previous studies of extant and ancestrally reconstructed proteins have revealed that DFI profiles correlate strongly with changes in thermodynamic and functional properties (*68*, *74*–*76*).

**Fig. 5.**
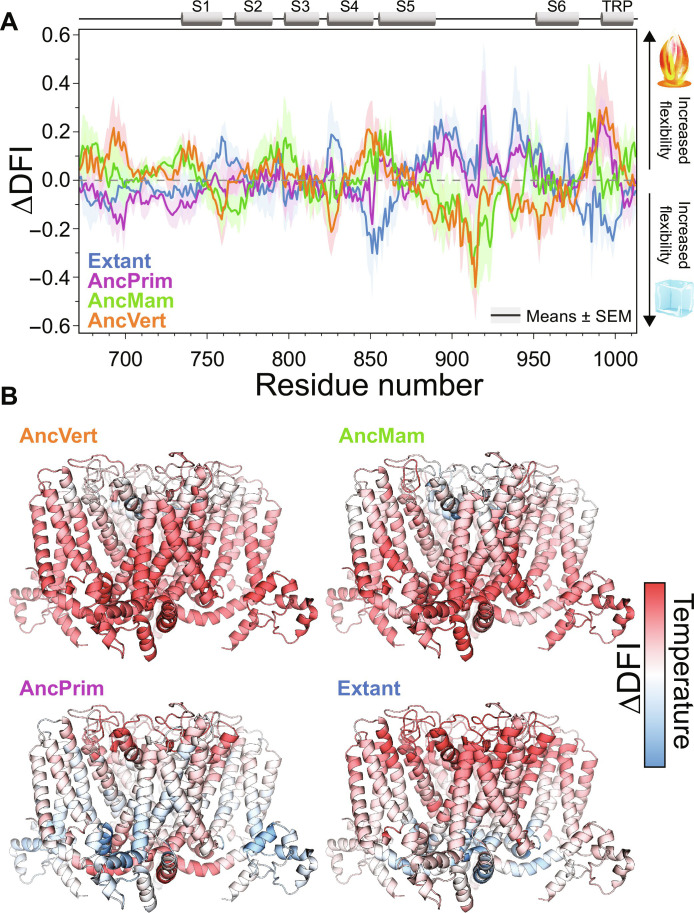
Temperature-dependent differences in calculated flexibility. (**A**) The difference between normalized DFI (ΔDFI) calculated at 27° and 10°C for extant, AncPrim, AncMam, and AncVert TRPM8 highlights regions with predicted temperature-dependent dynamics. The data represent the means ± SEM of the chain-wise ΔDFI. (**B**) The change in flexibility colored on the structure for all extant and ancestral TRPM8 orthologs. Regions colored red show increased flexibility on heating, and regions colored blue show increased flexibility on cooling.

We hypothesized that DFI might provide molecular details at different temperatures, with menthol and cold activation features dominant at ambient and low temperatures, respectively. To evaluate this hypothesis, we computed the DFI profile of the extant and ancestral TRPM8 TMD using data obtained from MD simulations performed at 27°C (300 K) and 10°C (283 K). Figure 5 shows the normalized difference in DFI (ΔDFI) at these temperatures. Principal component–based clustering of normalized DFI profiles of extant hTRPM8 and its ancestral nodes at 27°C recapitulates the hierarchy of menthol sensitivity identified functionally in Fig. 4 (fig. S14A). At 10°C, the DFI clustering analysis reflects the evolution of cold thermosensing identified in Fig. 3 (fig. S14B). To provide mechanistic insight into how TRPM8 cold sensing evolved, we computed ΔDFI in a temperature-dependent manner of extant and ancestral TRPM8 orthologs (Fig. 5A). ΔDFI analysis shows that increasing flexibility at low temperatures correlates with increased experimentally determined cold thermosensitivity. Mechanistically, extant hTRRPM8 activation and gating requires menthol or cold sensing to couple allosterically to the intracellular gate at the S6 helix bundle crossing. For the cold-sensitive extant and AncPrim TRPM8 orthologs, DFI predicts that regions of the VSLD, S6 gate, and TRP helix have increased flexibility at low temperature, consistent with our experimental results (Fig. 5B).

We note that normalized ΔDFI distribution variance provides insight into the thermodynamic stability of each variant. For the TRPM8 orthologs in this study, the ΔDFI variance distribution correlates moderately well (*R*^2^ = 0.87) with the experimentally determined melting temperatures from CETSA (fig. S15). The normalized ΔDFI correlations with menthol activation, cold thermosensitivity, and cellular stability support its further use to investigate the molecular mechanisms that underlie TRPM8 polymodal function specifically and other thermosensitivity and polymodal proteins in general.

## DISCUSSION

ASR is an orthogonal approach to study extant orthologs and was used to probe TRPM8 polymodal activation and evaluate the emergence of coupled ligand and cold activation modes. Evaluating extant TRPM8 ortholog studies to identify the origins of cold or menthol activation in TRPM8 evolution is complex. TRPM8 is reported to have emerged with vertebrate evolution (~600 to 550 Ma ago) (*13*, *86*). One hypothesis is that TRPM8 cold activation appeared first during the water-to-land transition of tetrapods (~400 to 350 Ma ago) (*87*, *88*). This is supported by TRPM8 ortholog phenotypes in extant aquatic animals, such as green sea turtles (*Chelonia mydas*) and caecilians (*Rhinatrema bivittatum*), which are not cold sensitive but are responsive to menthol (*87*). However, the African lungfish (*Protopterus annectens*) TRPM8 is neither cold nor menthol sensitive (*87*), suggesting that cold sensitivity may have emerged later than initially thought.

An alternative hypothesis is that menthol activation emerged first, perhaps as a way for early vertebrates to detect and avoid nocifensive terpenoid compounds produced by plants. Insect studies support this hypothesis, and while insects appeared well before vertebrates and lack TRPM8, they generally express a TRPM-like gene that is activated by cold and menthol (*89*–*91*). In the presence of menthol, fruit fly (*Drosophila melanogaster*) larvae respond by a TRPM-dependent nocifensive rolling behavior (*91*). Similarly, menthol-related terpenes, like carvone (spearmint oil), pulegone (pennyroyal oil), and menthone, have been shown to decrease the survival of fruit flies (*92*). Last, menthol is toxic to a variety of organisms, including fungi and bacteria, suggesting that TRPM8 activation by menthol may have played an important role in early chemical defense mechanisms (*93*–*95*).

**Fig. 6.**
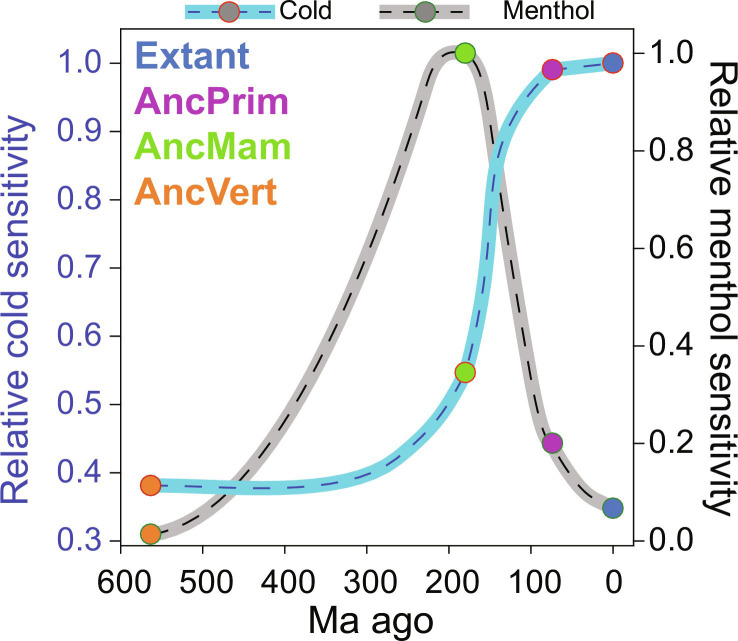
Relationship between TRPM8 thermosensitivity and menthol sensitivity with evolutionary time. An inverse relationship between cold (blue) and menthol sensitivity (gray) along the hTRPM8 evolutionary trajectory. ASR enables the dissection of TRPM8 polymodal activation. Data points are connected via spline interpolation.

Our data support that TRPM8 menthol activation emerged first, at least along the human evolutionary trajectory, peaking near AncMam TRPM8 (~200 Ma ago), and concurrently with decreasing menthol sensitivity (Fig. 6), modern cold activation emerged and was present in AncPrim TRPM8 (~75 Ma ago). Presumably, TRPM8 activation modes have evolved differently along distinct vertebrate lineages, which could convolute comparative extant studies and their interpretation. For example, some extant TRPM8 orthologs have diminished cold sensitivity, including some frogs (*Xenopus tropicalis* and *X. laevis*) (*41*) and hibernating rodents (*54*).

This study also highlights the general benefits of ASR. Single mutations are commonly associated with human diseases of either misfunction or mistrafficking. Accordingly, it is remarkable that, in a complex protein like TRPM8, functional reconstructed protein is accessible after tens to hundreds of mutations. For context, tetrameric AncVert has 496 mutations relative to extant hTRPM8. ASR sequences have long been used successfully to evaluate and decipher molecular mechanisms (*55*, *96*–*100*), and our DFI data (Fig. 5) show that this is the case for TRPM8 as well. Future studies will make use of the residue-specific DFI predictions to further delineate the TRPM8 mode-specific activation. Moreover, given our results in evaluating cellular trafficking and stability (Fig. 2), directed ASR studies could also help to decipher fundamental mechanisms of protein science, including the molecular origins of cellular trafficking and stability.

With regard to TRPM8, these studies enable us to partially deconvolute the coupling between cold and menthol activation modes and indicate that mutations spread throughout the channel (fig. S10) endowing hTRPM8 with exquisite cold sensitivity consistent with an emergent property. For TRPM8 and other thermosensitive TRP channels, species differences in channel function have been identified. Human and rodent TRPM8 orthologs have species-dependent regulation from natural compounds, synthetic antagonists, and modulatory proteins (*18*–*20*). For the better studied hot sensor TRPV1, species differences between rodent and human TRPV1 have been implicated in convoluting drug discovery issues (*16*, *17*, *51*).

An evolutionary study concluded that TRPM8 underwent two major diversification events, one in the Boreoeutheria ancestor (~100 Ma ago), that gave rise to both extant human and rodent TRPM8 lineages and a later diversification event after the divergence of ancestral rodent and hTRPM8 orthologs (*101*). Ancestral protein–based studies allow one to focus on the lineage of most interest, such as hTRPM8 in the case of drug discovery, which alleviates concerns arising from the noted species-dependent behavior. While TRPM8 has been implicated in contributing to unmet clinical needs, such as various pain syndromes, clinical trials targeting TRPM8 have generally elicited adverse side effects in thermal sensing and thermal regulation (*4*, *6*, *102*). Leveraging the functional differences in mode selectivity of extant and ancestral hTRPM8 orthologs, one could envisage developing side effect–free therapies by screening and counter-screening extant and ancestral proteins to identify the molecular origins of thermoneutral antagonists.

## MATERIALS AND METHODS

### Ancestral TRPM8 sequence reconstruction

To accurately estimate a phylogenetic tree and capture a comprehensive understanding of evolutionary information, it is essential to analyze a diverse range of species (*25*). For this purpose, we collected various orthogonal extant TRPM8 species sequences from different sources. We constructed three separate phylogenetic trees for better analysis: (i) the first comprised 32 sequences from 46 vertebrate species with a time period of about ~600 Ma and accurately determined divergence times (data S1) (*25*); (ii) the second list consisted of 21 TRPM8 species selected to investigate the molecular evolution and elucidate selection pressure on critical regions of the protein (data S2) (*86*); and (iii) the third list involved a diverse collection of 250 species gathered, using a protein Basic Local Alignment Search Tool with a BLOSUM62 substitution matrix on homologous sequences from National Center for Biotechnology Information Protein Reference Sequences, setting an *E*-value threshold of 0.005 and a minimum sequence identity of 70% (data S3) (*103*, *104*). For each species set, the harvested TRPM8 sequences underwent multiple sequence alignments (MSA) using MUltiple Sequence Comparison by Log-Expectation (MUSCLE) (*105*). The MSA results for each species set are provided as supplementary data files. To construct phylogenetic trees, molecular phylogeny analysis was used by using Molecular Evolutionary Genetics Analysis (MEGA) software with the computed MSAs (*106*, *107*). The maximum likelihood method was used to optimize tree topology, branch lengths, and tree models. The initial trees were generated using the neighbor-joining method on the pairwise distance matrix estimated by the Jones-Taylor-Thornton substitution model with an evolutionary rate model selected as uniform distribution. The predicted TRPM8-based phylogenetic tree with the highest log-likelihood value was chosen for each subset (fig. S1). MEGA software was also used to estimate and reconstruct ancestral TRPM8 sequences (*25*, *106*). The amino acid position at each branch node was treated as a variable and identified the maximum probability of identity given the input sequence and the constructed phylogenic tree data. TRPM8 ancestral consensus sequence nodes for primate, mammalian, and vertebrate ancestors were constructed using the MEGA software (fig. S2). The evolutionary timescale estimates of divergence times for primates, mammalia, and vertebrata were obtained from TimeTree5 (*108*). The alignment of the consensus sequences is shown in fig. S3, providing a comprehensive analysis of various TRPM8 species and their evolutionary relationships.

### Molecular biology

The AncPrim, AncMam, and AncVert *TRPM8* genes were synthesized by Biomatik, flanked by Xho I and Bam HI restriction sites in a pBluescript II Sk(+) vector. The ancestral *TRPM8* genes were subcloned into a bicistronic plasmid with an internal ribosome entry site element and an enhanced green fluorescent protein gene (pIRES2-EGFP) plasmid via restriction digestion and ligation for electrophysiology and flow cytometry experiments. We have previously extensively characterized the pIRES2-hTRPM8-EGFP vector (*18*, *19*, *31*, *109*). The resulting pIRES2-TRPM8-EGFP plasmids were modified to insert an N-terminal hemagglutinin (HA) epitope using a Q5 mutagenesis kit [New England Biolabs (NEB)] for CETSA experiments as described below. The extant hTRPM8 construct with an N-terminal HA epitope was previously made and characterized (*18*).

The extant and ancestral *TRPM8* genes and a compatible pKanAttb_IRES-mCherry-H2A-P2A-PuroR backbone were amplified by polymerase chain reaction (PCR). HiFi assembly (NEB) was used to generate pKanAttB_TRPM8_IRES-mCherry-H2A-P2A-PuroR plasmid for each TRPM8 construct. The pKan plasmids were used in conjunction with HEK-293T Bxb1 landing pad recombination to generate stably expressing extant and ancestral cells for calcium flux assays (described below). All PCR primers are listed in table S7.

### Single-cell trafficking assay

The comparison of cell surface trafficking of ancestral and extant TRPM8 was done through single-cell live cell flow cytometric analysis. HEK-293 cells [American Type Culture Collection (ATCC), CRL-1573] were grown in Dulbecco’s modified Eagle’s medium (DMEM) (Gibco, 11960077) supplemented with 10% fetal bovine serum (FBS; Gibco, 16000), 2 mM l-glutamine (Gibco, 25030), and penicillin-streptomycin (100 U/ml; Gibco, 15140) maintained at 37°C with 5% CO_2_. HEK-293T cells stably expressing TRPM8 (a gift from T. Voets by way of V. Journigan) were grown in DMEM supplemented with 10% FBS, 4 mM l-glutamine, penicillin-streptomycin (100 U/ml), 100 μM nonessential amino acid solution (Gibco, 11140050), 4 mM GlutaMAX (Gibco, 35050061), G418 (200 μg/ml; Sigma-Aldrich, A1720), and 0.12% sodium bicarbonate (Gibco, 25080094) and maintained at 37°C with 10% CO_2_. The stable TRPM8-expressing cells were used as a positive control for plasma membrane trafficking. For other trafficking studies, transient transfection was used with a pIRES2-EGFP vector. HEK-293 cells were plated onto two 60-mm dishes and allowed to reach 50 to 60% confluency and then transfected with 4 μg of maxi-prepped DNA containing either the extant or ancestral *TRPM8* gene, or empty vector negative control, with 12 μl of polyethyleneimine (PEI) (1 mg/ml; 25 kDa, linear; Polysciences, 23966) incubated in 500 μl of serum-free DMEM for 30 min at room temperature. Cells were prepared for analysis ~46 hours after transfection.

Dishes were washed with 1 ml of warm phosphate-buffered saline (PBS; Gibco, 10010031) for 1 min at 37°C, and cells were triturated with 1 ml of fluorescence-activated cell sorting (FACS) buffer (PBS with 2% FBS). Following filtration through a 30-μm mesh (CellTrics, 04-004-2326), cells were spun down at 100*g* for 2 min at 4°C. Cells were then resuspended in 100 μl of FACS buffer with 2 μl of α-TRPM8 (C-940) rabbit polyclonal antibody (Abcepta, AP8181D) and incubated on ice for 1 hour with gentle agitation every 15 min. Cells were then washed five times with 1 ml of FACS buffer and resuspended in 100 μl of FACS buffer with 0.5 μl of α-rabbit Alexa Fluor 647 (AF647) goat polyclonal antibody (Invitrogen, A-21244). Cells were then incubated on ice in the dark for 1 hour with gentle agitation every 15 min. Cells were washed five times with 1 ml of FACS buffer and resuspended in 500 μl of FACS buffer for quantification on a four-laser Thermo-Fisher Attune NxT. Forward and side scatter voltages were set using untransfected HEK-293 cells, EGFP voltage was set using empty vector–EGFP-transfected cells, and AF647 voltage was set using HEK-293T cells stably expressing TRPM8.

Ungated cell populations (Fig. 2A) were plotted using MATLAB, with empty vector–EGFP-transfected cells and stable TRPM8-expressing cells being used as positive controls for transfection and surface TRPM8 expression, respectively (fig. S4). MATLAB plots were generated by modified code of Henson (*110*). For cells determined to be EGFP positive (gated using untransfected HEK-293 cells), the ratio of AF647 (used to stain cell surface TRPM8) to EGFP (the transfection marker) was used as a corollary for relative trafficking between constructs normalized by expression. These ratios were compared using an unpaired *t* test.

### Cellular thermostability measurements

CETSAs were conducted on the basis of the previous studies (*18*, *47*–*49*). HEK-293 cells (ATCC, CRL-1573) were cultured in DMEM supplemented with 10% FBS, penicillin-streptomycin (100 U/ml), and 2 mM l-glutamine (Gibco) at 37°C in the presence of 5% CO_2_ in 100-mm dishes. The cells were transfected with N-terminally HA-tagged extant and ancestral *TRPM8* genes in a pIRES2-EGFP vector. Specifically, 10 μg of plasmid DNA was incubated at room temperature containing 30 μl of PEI MAX and 1 ml of serum free DMEM before adding to the dishes. Our previous studies show that N-terminally HA-tagged TRPM8 functions normally (*18*). While on ice and 42 to 48 hours after transfection, HEK-293 cells were washed three times with cold PBS buffer (pH 7.4) to remove residual medium before incubating with 500 μl of cold 50 mM tris, 150 mM NaCl, 5 mM EDTA, and Pierce protease inhibitor mini tablets (Thermo Fisher Scientific, no. A32953) (pH 7.4) for about 1 min. Cells were then harvested using a cell scraper and evaluated using Countess 3 FL to count cell numbers and quantify transfection efficiency. Cells were divided into 50-μl aliquots containing roughly 1.5 × 10^6^ to 3.0 × 10^6^ cells/ml. Sample heating was carried out in a Bio-Rad T100 PCR thermocycler. The samples were warmed to 25°C for 30 s before ramping to the desired temperature and between 37° and 58°C for 3.5 min followed by cooling to 4°C. Cells were lysed by adding 1% Nonidet P-40 substitute (4-nonylphenyl–polyethylene glycol), 0.5% sodium deoxycholate, and 0.1% SDS and tumbling for 15 min at 4°C. Precipitants and insoluble components were separated by centrifugation at 14,000*g* for 10 min at 4°C. The supernatant was collected and mixed with 6× SDS–polyacrylamide gel electrophoresis loading dye. Samples were analyzed using Western blot and probed with a mouse monoclonal anti-HA primary antibody (Thermo Fisher Scientific, catalog no. 26183) diluted to 1:1500 and horse anti-mouse immunoglobulin G horseradish peroxidase–linked secondary antibody (Cell Signaling Technology, catalog no. 7076) was diluted to 1:2000. Blots were developed with Clarity Enhanced Chemiluminescence Western Blotting Substrates (Bio-Rad) according to the manufacturer’s protocol. Blots were imaged directly using a Nikon D610 DSLR camera and Nikkor 50-mm f/1.4G lens and processed using Photoshop, and band intensities were analyzed using ImageJ (*18*, *111*). To minimize experimental bias, CETSA was conducted in four to five independent biological sets of experiments that were carried out on independently transfected dishes of cells on different days.

### Cell culture for electrophysiology measurements

Cell culture was conducted as previously (*18*, *31*, *50*, *109*). HEK-293 cells (ATCC, CRL-1573) were cultured in DMEM supplemented with 10% FBS, penicillin-streptomycin (100 U/ml), and 2 mM l-glutamine (Gibco) at 37°C in the presence of 5% CO_2_. Cells were cultured in 35- or 60-mm dishes (Falcon) and split every 2 to 3 days. Cells were transiently transfected with 0.5 μg of plasmid DNA, containing the AncM8s in a pIRES2-EGFP vector, and 1.5 μl of FuGENE 6 transfection reagent (Promega) in 100 μl of serum-free DMEM. The transfection mixture was incubated for 30 min at room temperature before adding to the cells. Following 36 to 48 hours after transfection, cells were seeded onto glass coverslips after incubated with 0.25% trypsin/EDTA and resuspended in medium and allowed to recover for 1 to 2 hours at 37°C, 5% CO_2_, before conducting whole-cell patch-clamp experiments.

### Whole-cell patch-clamp electrophysiology

Whole-cell patch-clamp current measurements were performed using an Axopatch 200B amplifier (Axon Instruments) and pClamp 10.3 software (Axon Instruments), and experiments were recorded at 2 kHz and filtered at 1 kHz. Cells are placed on glass coverslips with extracellular buffer of 10 mM Hepes, 132 mM NaCl, 4.8 mM KCl, 1.2 mM MgCl_2_, 2 mM CaCl_2_, and 5 mM glucose (pH 7.4). The extracellular buffer pH was adjusted using NaOH, and osmolality was adjusted to 310 mosmol with sucrose. Osmolality was measured using a Vapro 5600 vapor pressure osmometer (Wescor). Pipettes were fabricated with a P-2000 laser puller (Sutter Instruments) from borosilicate glass capillaries (World Precision Instruments). These were heat-polished using a MF-830 microforge (Narishige) and had a resistance of 2 to 5 megaohms. The glass pipettes were then filled with an intracellular buffer of 10 mM Hepes, 135 mM potassium gluconate, 5 mM KCl, 1 mM MgCl_2_, and 5 mM EGTA (pH 7.2). The pH was adjusted using KOH, and osmolality was adjusted to 300 mosmol using sucrose. The ground electrode was inserted into 2% agar bridge made from the extracellular buffer. Experiments were performed at 23 ± 1°C unless otherwise noted. Menthol was dissolved in dimethyl sulfoxide (DMSO) before diluting to desired concentrations with extracellular solutions. Temperature was controlled using a Peltier-based perfusion system (ALA Scientific). For cold experiments, 2-min recordings were done while perfusing in a temperature ramp of 28° to 8°C at 60 mV. For heat experiments, 8-s recordings were done at either 25° or 40°C. To maximize the reproducibility and quantitative nature of the electrophysiology experiments, we use epifluorescence to identify candidate TRPM8-expressing cells with similar EGFP intensity. In addition, we measure the cell capacitance, which is a proxy for cell size, and normalize the currents by this value, giving rise to current density values (picoamperes per picofarad).

### Generation of Bxb1 lentiviral landing pad stable HEK-293T cells for calcium mobilization assay

HEK-293T lentiviral landing pad cells (*112*) were transfected with 0.5 μg of a modified pLenti-TetBxb1BFP-Int (LLP-Int) plasmid that includes either extant or ancestral *TRPM8* genes with 1.5 μl of FuGENE6 in serum-free DMEM after allowing the transfection mixture to incubate 30 min at room temperature. Cells were cultured in DMEM supplemented with 10% FBS, penicillin-streptomycin (100 U/ml), and 2 mM l-Gln (Gibco) at 37°C in the presence of 5% CO_2_. Two days after transfection, the medium was supplemented with doxycycline (2 μg/ml) to initiate Bxb1 DNA recombination. Four days after transfection, the medium was supplemented with puromycin (1 μg/ml) for positive selection recombinant cells. The selection process was monitored by fluorescence using a Countess 3 FL automated cell counter with 4′,6-diamidino-2-phenylindole, Texas Red, and red fluorescent protein (RFP) light cubes. About 2 weeks after transfection, cells were used for calcium mobilization experiments after reaching ≥85% red fluorescence (positive recombinant) and ≤ 10% blue fluorescence (nonrecombinant cells).

### Calcium mobilization assay

Tissue-culture–treated black 96-well plates with clear flat bottom were treated with poly-l-ornithine (20 μg/ml) for 30 min before washing twice with 1× PBS (pH 7.4). Approximately 50,000 cells were added to each well and allowed to incubate overnight at 37°C with 5% CO_2_. The medium was discarded, and the wells were washed with Hanks’ balanced salt solution (HBSS) containing 1.2 mM CaCl_2_, 4.9 mM MgCl_2_, 4 mM MgSO_4_, 53.3 mM KCl, 4.4 mM KH_2_PO_4_, 3.4 mM Na_2_HPO_4_, 1.38 M NaCl, and 20 mM Hepes (pH 7.2). Fluoforte (Enzo Life Sciences) was added and incubated for 1 hour at room temperature in the dark. Following the incubation, the dye was removed, and the cells were washed twice with the HBSS buffer. The cells recovered for 10 min at room temperature in the dark before the plate was scanned using Tecan Spark multimode plate reader. The reagent (20 μl) was injected into each well after 5 s of background reading and continued to record for an additional 40 s at 100 gain and 25°C. Menthol solutions were made by dissolution in DMSO and further serial dilutions in the HBSS buffer.

### MD simulation

Homology models of the TMD of hTRPM8 were generated using Rosetta comparative modeling. The electron density map from the avian *Ficedula albicollis* TRPM8 (*Fa*TRPM8, 6BPQ) cryo–electron microscopy structure and TRPM4 were used as templates. Loops and side chains that are present in hTRPM8 but absent in *Fa* TRPM8 were modeled using implicit membrane potentials (*35*). The obtained models of ancestral proteins were then used to create structural predictions for the ancestral sequences by using the PyMOL mutagenesis tool (*113*). To evaluate the modeling robustness, we also generated homology models from AlphaFold (fig. S16) (*114*). AlphaFold models gave relatively high confidence scores (>82), and alignment with the Rosetta-based models used for MD simulations below gave reasonably low Cα root mean square deviation values over the transmembrane helices. We note, and as expected, that loop regions were the largest source of model differences between the Rosetta- and AlphaFold-based models, with the most substantial differences occurring between the S5 and S6 loops (fig. S16).

The ancestral variant models along with the model for the hTRPM8 were then embedded in a membrane composed of 1-palmitoyl-2-oleoyl-*sn*-glycero-3-phosphocholine in a 1:1 ratio. To neutralize the charge of the system, sodium and chloride ions were added at a concentration of 0.15 M. The simulations were then performed for all the variants using the CHARMM36m (*115*) force field for both lipid and protein. The CHARMM-GUI Membrane Builder (*116*) was used to assemble the system and generate the AMBER 20 input files. We then performed an energy minimization and allowed the system to equilibrate using the standard six-step CHARMM-GUI protocol (*117*). Two sets of constant number of particles, pressure, and temperature (NPT) simulations were done with a time step of 2 fs for the extant, and each ancestral variant with temperatures was maintained at 300 and 283 K using a Langevin thermostat. The pressure was maintained at 1 bar using a Monte Carlo barostat. The simulations were performed using the graphics processing unit (GPU)–accelerated particle mesh Ewald molecular dynamics (PMEMD) module of AMBER 20 (*118*) in a water box of dimension of 140 Å by 140 Å by 98 Å with water thickness (amount of water added above and below the membrane) set to 14 Å and periodic boundary conditions using the TIP3P water model. The sampled conformations were saved every 50 ps, and each simulation was run for over 2 μs to give the systems enough time to converge. The convergence analysis was then performed (*119*).

### Computational dynamical analysis

The dynamical stability of each residue position was calculated using the DFI (*70*, *74*, *80*, *120*). The DFI provides important information about protein function by quantifying the resilience of each residue to perturbations at every other site. For example, residues that have a low DFI score are known as hinge sites and are typically found to be evolutionarily conserved as they play a key role in transferring the perturbation energies through the protein (*73*, *80*, *81*, *120*, *121*).

This metric is based on the PRS method (*122*). Here, the Cα atom of each residue is modeled as a node in an elastic network model (*123*), where interactions between each node are modeled using a harmonic potential. For such a model, the effect of a perturbation can be obtained using LRT as

$${\left[\Delta R\right]}_{3N\times 1}={\left[H\right]}_{3N\times 3N}^{-1}{\left[F\right]}_{3N\times 1}$$

where **F** is the random force, **H** is the Hessian, and **ΔR** gives the positional response of each residue. To take into account all the long-range interactions, solvation effects, and biochemical specificities and study the effects of mutations on the dynamics, we can replace the Hessian inverse with the covariance matrix **G** that we obtain from MD simulation trajectories (*69*, *81*, *121*, *124*). Thus, the response now becomes

$${\left[\Delta R\right]}_{3N\times 1}={\left[G\right]}_{3N\times 3N}{\left[F\right]}_{3N\times 1}$$

To determine the DFI, each perturbation was calculated along seven different directions to ensure an isotropic response. We can determine the perturbation matrix **A** by calculating the response of each position on perturbing every other position. This can then be written as

$${\left[A\right]}_{N\times N}=\left[\begin{array}{ccc} {|{\Delta R}^{1}|}_{1} & \cdots & {|{\Delta R}^{N}|}_{1}\\ \vdots & \ddots & \vdots \\ {|{\Delta R}^{1}|}_{N} & \cdots & {|{\Delta R}^{N}|}_{N} \end{array}\right]$$

where ${|{\Delta R}^{j}|}_{i}=\sqrt{<{\left(\Delta R\right)}^{2}>}$ gives the response at residue *i* when *j* is perturbed.

The DFI can then be calculated as

$$\text{DF}I_{i}=\frac{\sum_{j=1}^{N}{|\Delta R^{j}|}_{i}}{\sum_{i=1}^{N}\sum_{j=1}^{N}{|\Delta R^{j}|}_{i}}$$

The denominator used to normalize this metric gives us an idea of the overall response of the protein to perturbation. Combining MD-based residue covariances with force perturbation allows us to detect the dynamic protein motions as they propagate through the three-dimensional protein structure; incorporating force as an additional probe allows us to go beyond analyses of mean square fluctuations and capture couplings between amino acids (*69*, *70*). Thus, DFI computes the normalized response of each position to perturbations at all other positions and recapitulates the per-residue relative contribution to the conformational entropy of the protein.

To exactly quantify the flexibility of each residue in the protein, it is often useful to perform a percentile ranking on the computed DFI profile. The %DFI can be determined using

$$\%\text{DF}I_{i}=\frac{n_{\leq i}}{N}$$

where *n*_≤*i*_ represents the number of residues with DFI ≤ DFI*_i_*. Because TRPM8 is tetrameric, we calculated the percentile ranking for each protomer separately and then averaged them.

In our simulations, we calculated the effect of temperature on the dynamics of the system and defined the ΔDFI as

$$\Delta \%\text{DFI}=\%\text{DF}I_{300K}-\%\text{DF}I_{283K}$$

To calculate the DFI for each variant, we obtained the covariance matrices from the MD trajectories. This was done using AMBER’s cpptraj module over 100-ns moving windows that overlap by 50 ns. The analysis was performed using only the last 500 ns of the ≥1.5-μs individual trajectories to ensure that the covariance matrices accurately capture the equilibrium coordinate information and are independent of the initial atomic coordinates.

## Supplementary Materials

### This PDF file includes:

Figs. S1 to S16 Tables S1 to S7 Legends for data S1 to S4 References
