# Supplementary material for: Evidence that the cold- and menthol-sensing functions of the human TRPM8 channel evolved separately
Source: Sci Adv. 2024 Jun 21;10(25):eadm9228. doi: 10.1126/sciadv.adm9228 (PMC11192081; doi:10.1126/sciadv.adm9228)
Supplement: Supplementary file 1 — Figs. S1 to S16 Tables S1 to S7 Legends for data S1 to S4 References [file sciadv.adm9228_sm.pdf]

Science Advances
AAAS

Supplementary Materials for

Evidence that the cold- and menthol-sensing functions of the human TRPM8 channel evolved separately

Dustin D. Luu *et al.*

Corresponding author: Wade D. Van Horn, wade.van.horn@asu.edu; S. Banu Ozkan, banu.ozkan@asu.edu

Sci. Adv. **10**, eadm9228 (2024)
DOI: 10.1126/sciadv.adm9228

The PDF file includes:

Figs. S1 to S16
Tables S1 to S7
Legends for data S1 to S4
References

Other Supplementary Material for this manuscript includes the following:

Data S1 to S4

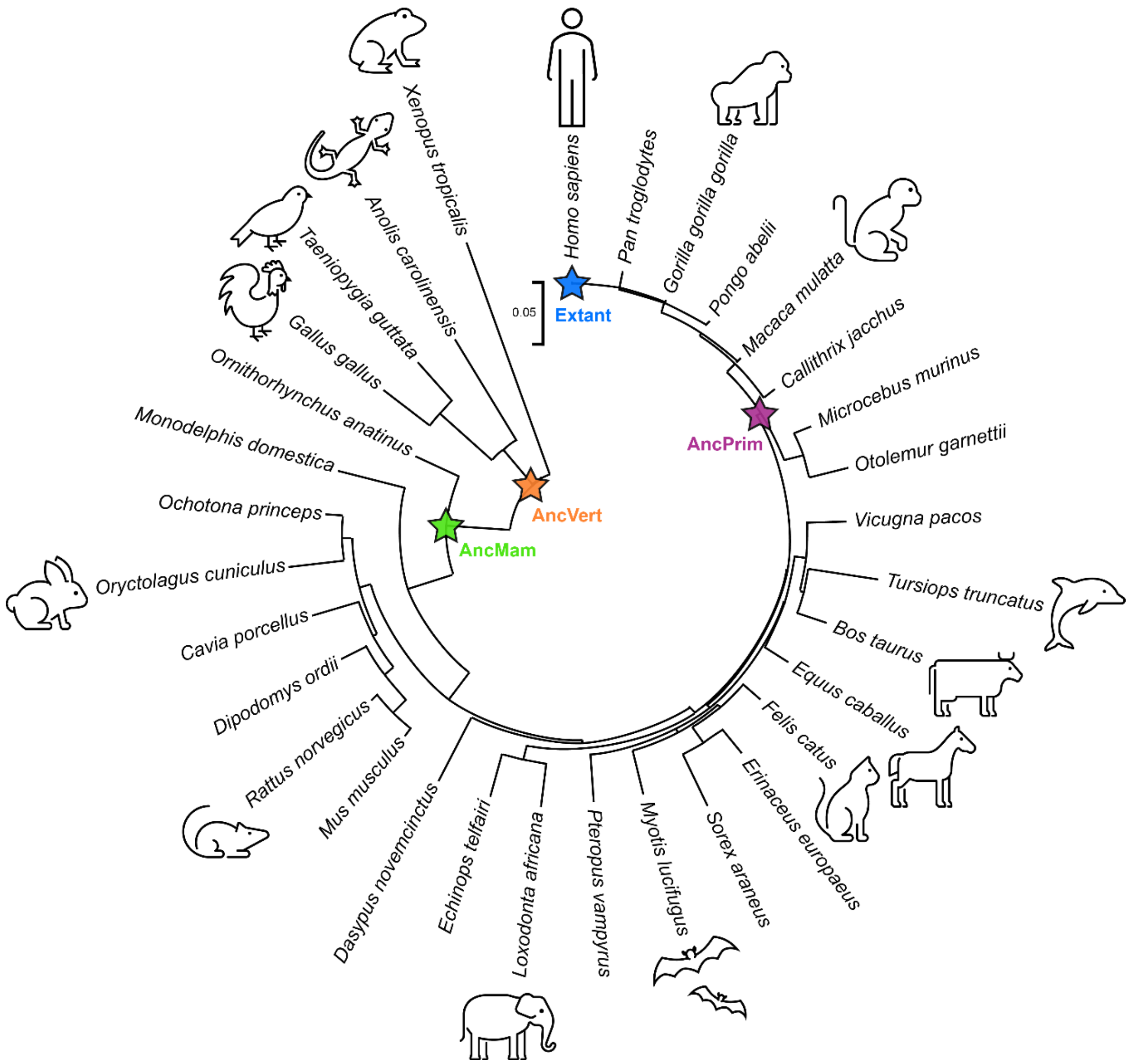

Fig. S1. TRPM8-constructed evolutionary timetree. Evolutionary timetree from one of the three sets derived exclusively from 32 vertebrate species of TRPM8 sequences and constructed with MEGA software (*107*). The reconstructed ancestral TRPM8 nodes studied herein are shown (stars) with extant (blue), AncPrim (purple), AncMam (green), AncVert (orange). The line distance reference to the percentage of genetic difference.

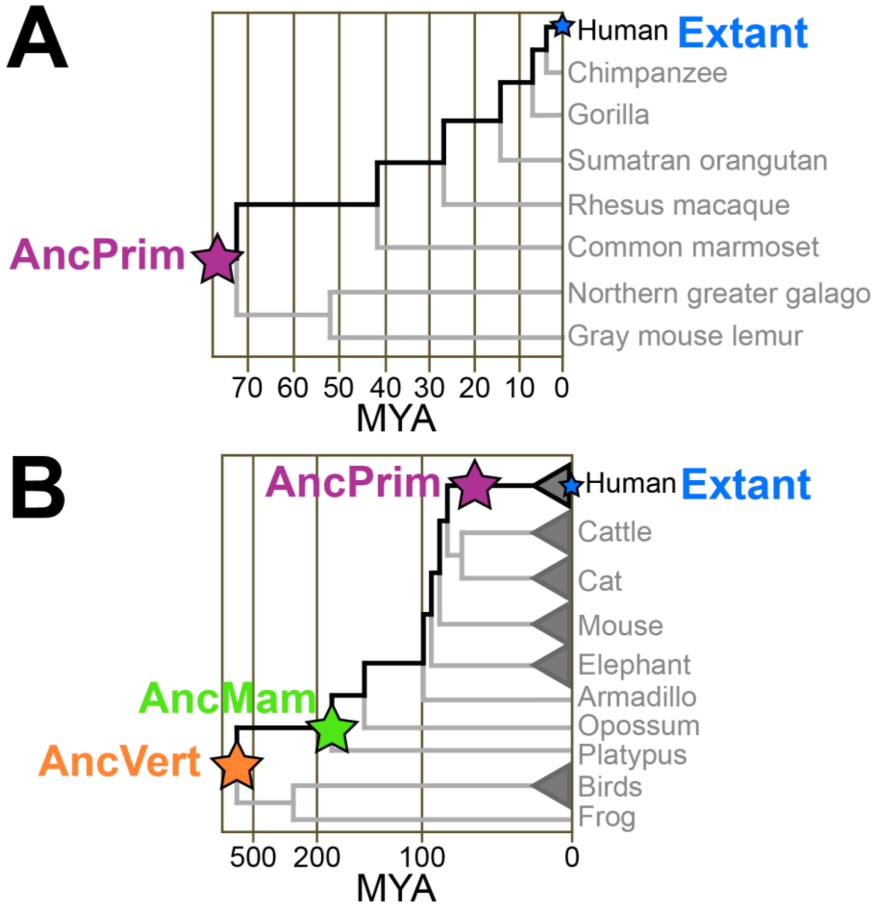

Fig. S2. Key nodes and phylogenetic analysis of ancestral TRPM8 along the human evolutionary trajectory. Details of the constructed phylogenetic tree and the nodes the reconstructed ancestral TRPM8 nodes are shown (stars). (**A**) Ancestral primate (AncPrim) sequence spans back over ~75 million years ago and encloses sequences from Human to Lemurs. (**B**) The nodes of the tree coinciding with ancestral mammalian (AncMam) and vertebrate (AncVert) reconstructions are shown. Divergence times are not to scale.

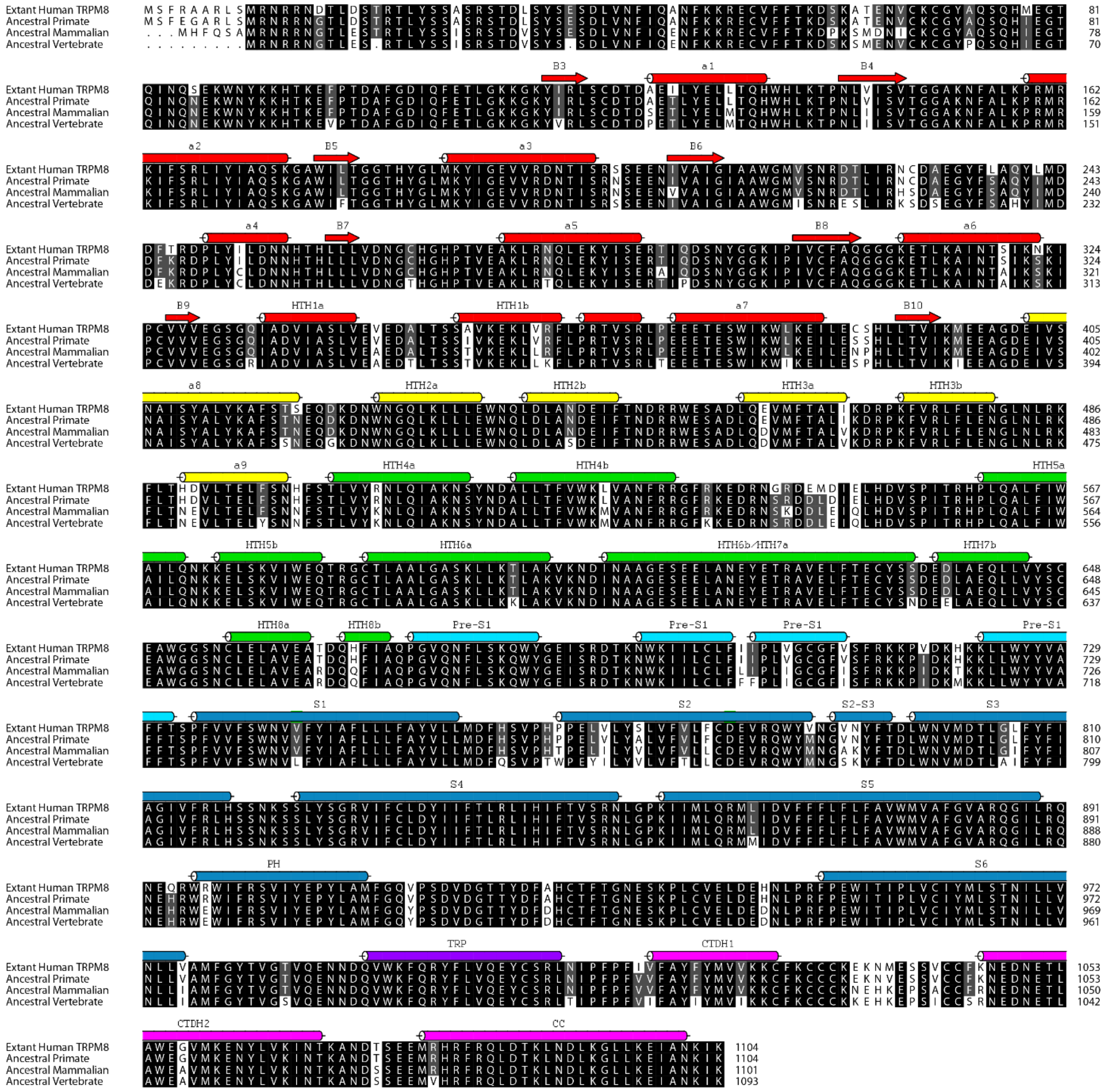

Fig. S3. Alignment of extant human and ancestral TRPM8 protein sequences. The secondary structures from flycatcher (*37*) and mouse (*34*) TRPM8 cryo-EM structures are indicated as cylinders (helices) and arrows (β-strands) with different regions colored based on the two TRPM8 cryo-EM structures. Sequences in black with white lettering show are conserved. Gray highlights with white lettering designate a single variance difference between orthologs. White with black lettering shows two or more variance differences between the ortholog sequences. The alignment was made by Clustal Omega (*125*) and displayed in ALINE (*126*).

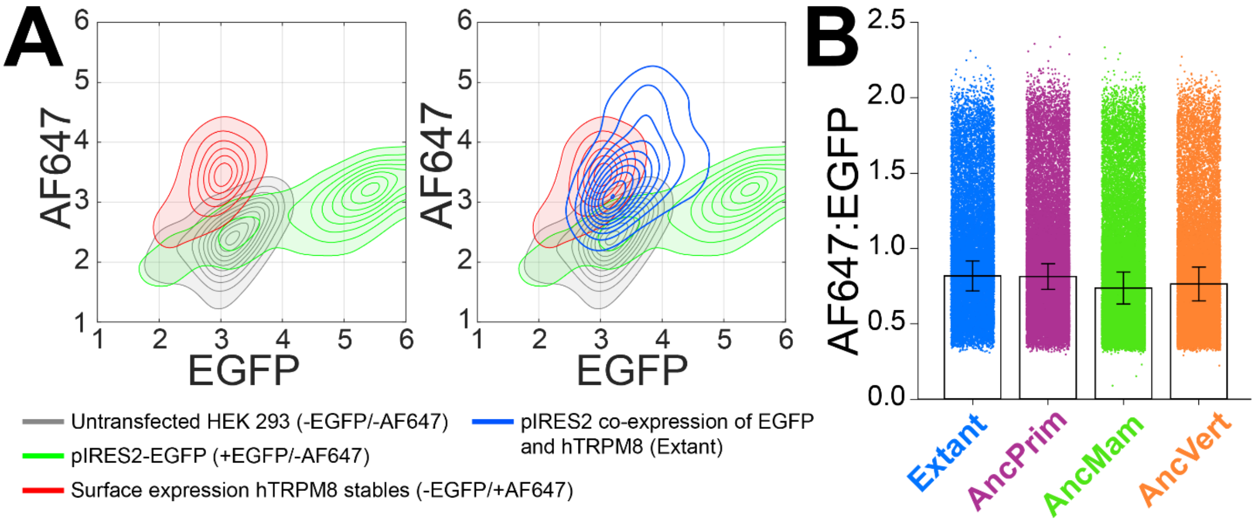

Fig. S4. Control experiments for flow cytometry trafficking to the plasma membrane. (**A**) Flow cytometry contour plots in logarithmic scale showing the fluorescence intensity distribution with enhanced green fluorescent protein (EGFP) and Alexa Fluor 647 (AF647). Untransfected HEK-293 cells (grey) were used as a negative control for both EGFP and AF647. HEK-293 cells transfected with pIRES2 vector with EGFP (pIRES2-EGFP, green) were used as a control for pIRES2 co-expression of EGFP and hTRPM8 (Extant, blue). HEK-293T stably expressing human TRPM8 (Surface expression hTRPM8 stables, red) was used to indicate surface expression of hTRPM8 by using anti-TRPM8 rabbit polyclonal antibody with anti-rabbit AF647 goat polyclonal secondary antibody. The cells used to make the contours were ungated and normalized to the lowest cell count of 4203. (**B**) The average ratio of AF647:EGFP from EGFP+ cells for Extant, AncPrim, AncMam, and AncVert. The averages are from six biological replicates conducted on different days. The jitter shown is the AF647:EGFP of individual cells from one of the replicates with 17843, 24269, 27388, and 21234 cells for Extant, AncPrim, AncMam, AncVert, respectively.

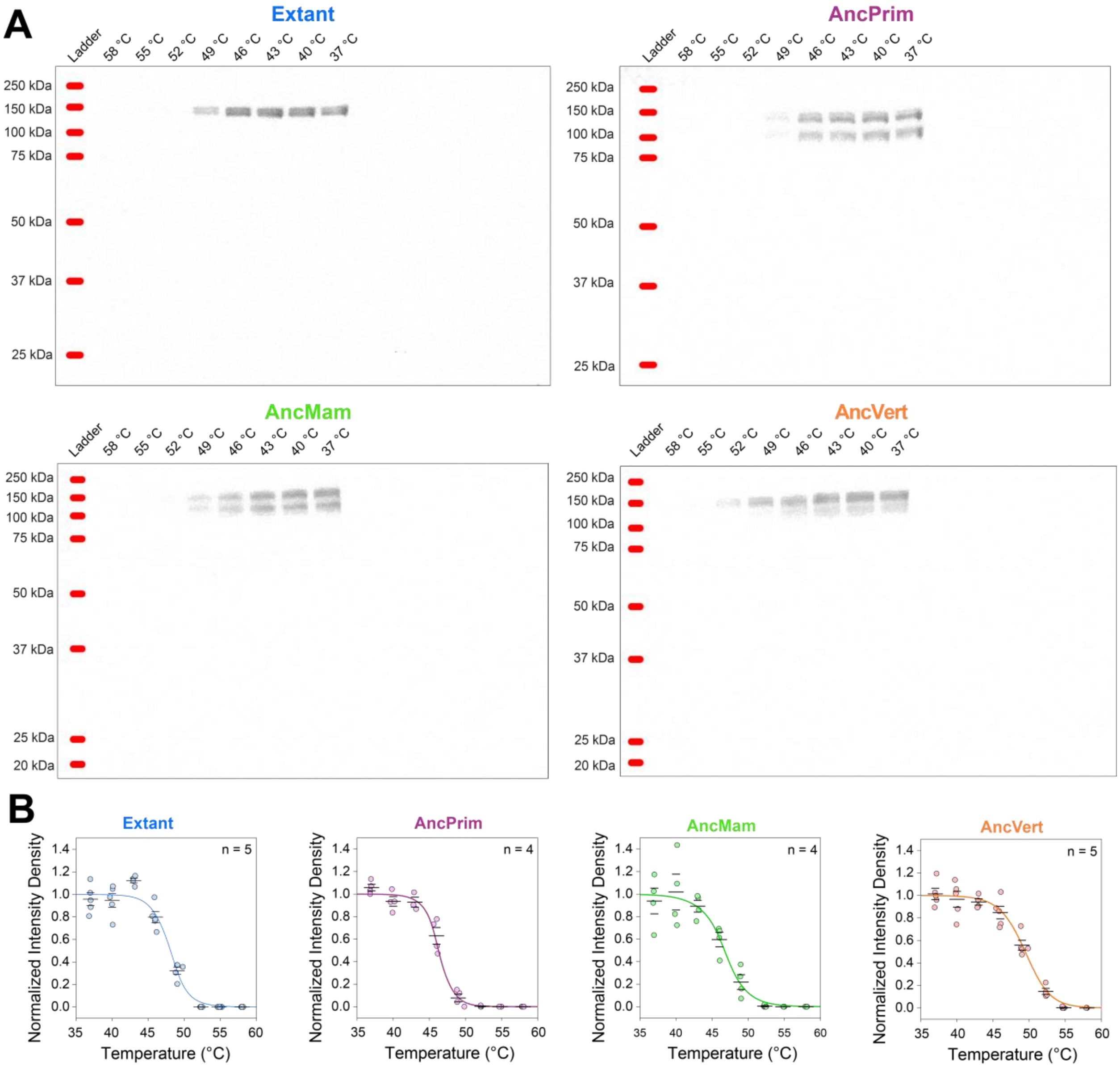

Fig. S5. CETSA western blot data and analysis of extant human and ancestral TRPM8 cellular thermal stability. (**A**) Representative western blots of the extant and ancestral TRPM8 orthologs. Mouse monoclonal anti-HA primary antibody with a horse anti-mouse IgG HRP-linked secondary antibody was used. The top bands were used to calculate the T_{50} and enthalpy values. No difference in melting temperatures were seen when both top and bottom bands were used for analysis. (**B**) Thermal stability curves with mean (middle band) and SEM (top and bottom band) at each temperature with jitters of each experiment. Biological replicates were done in 5, 4, 4, and 5 for extant, AncPrim, AncMam, AncVert, respectively.

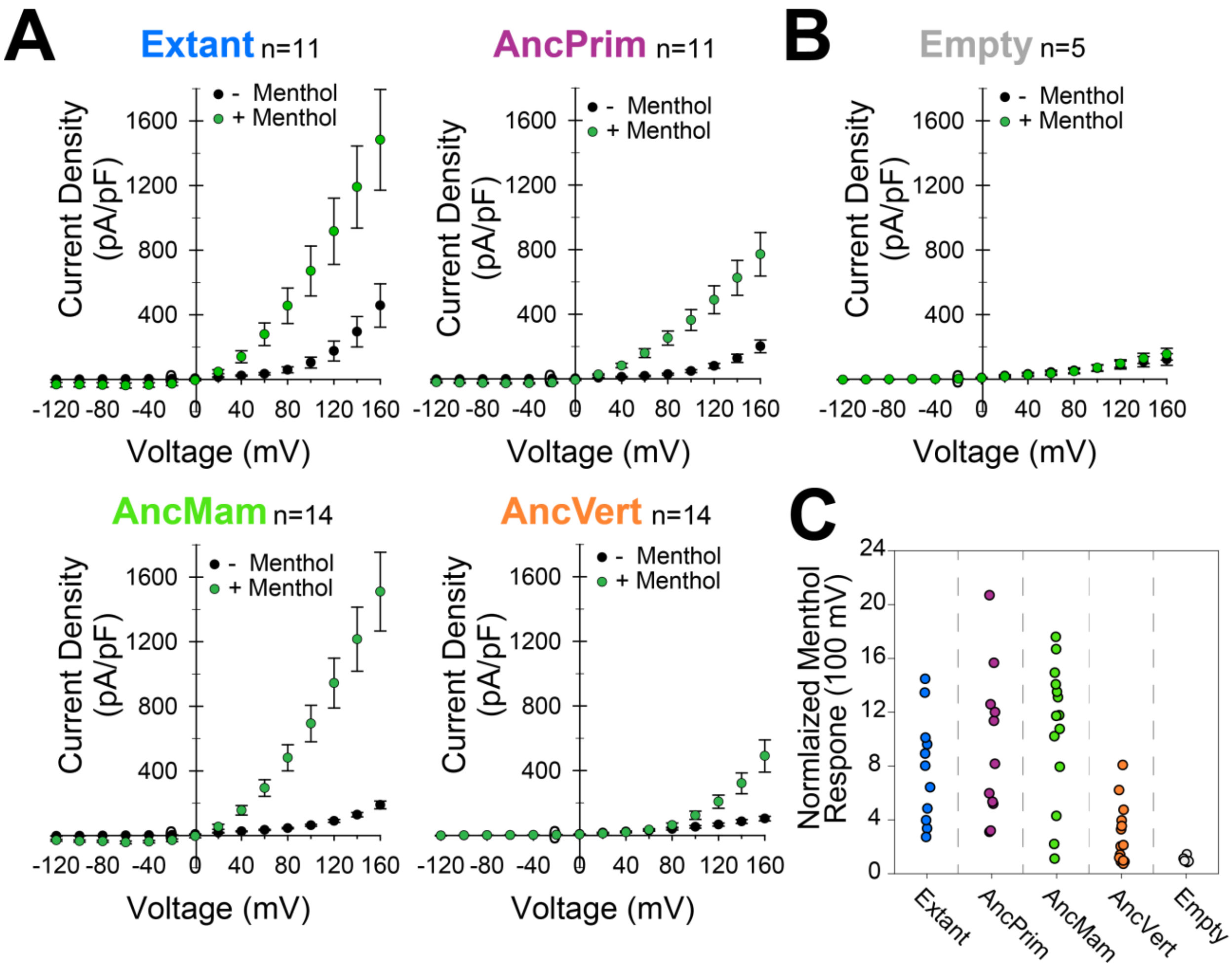

Fig. S6. Electrophysiology data show ancestral TRPM8 orthologs are menthol-activated. (**A**) IV plots with (green) and without (black) 500 µM menthol of the Extant, AncPri, AncMam, and AncVert from biological replicates of 11, 11, 14, and 14, respectively. Errors are in standard error of the mean. (**B**) Empty pIRES2 vector control with and without 500 µM menthol from biological replicates of 5. Errors are in standard error of the mean. (**C**) Jitter plot of the individual normalized menthol response

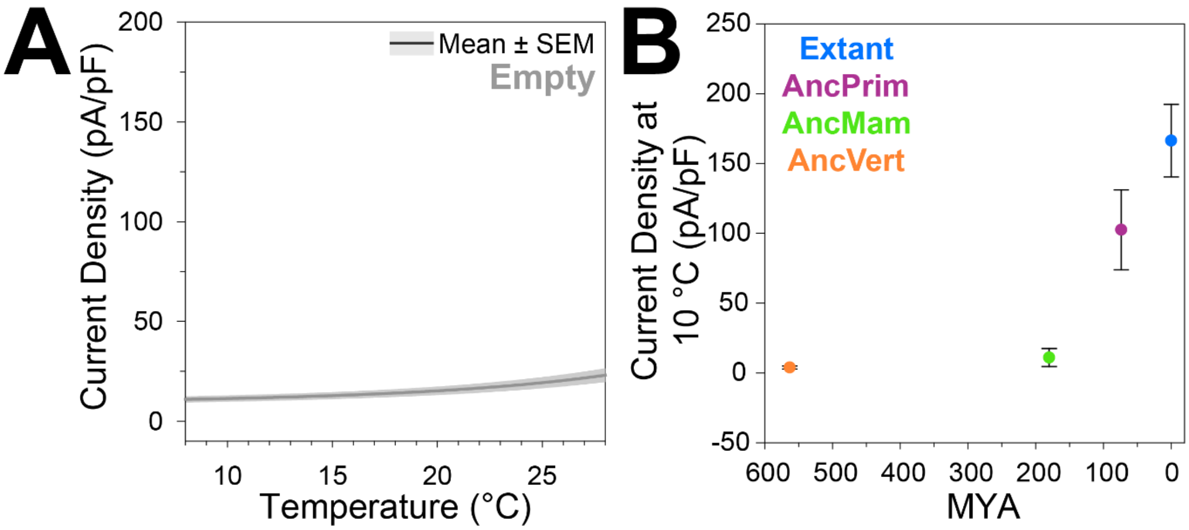

Fig. S7. **Thermosensitivity of empty pIRES2 control and extant human and ancestral TRPM8 orthologs as a function of evolutionary time.** (**A**) Whole-cell patch clamp electrophysiology measurements of the empty pIRES2 vector current density exposed to a temperature ramp from 28 °C to 8 °C. The data is from an average of 9 biological replicates. (**B**) Cold stimulated current density with standard error of the mean measured at 10 °C from decreasing temperature ramps (Fig. 3) are plotted against evolutionary time.

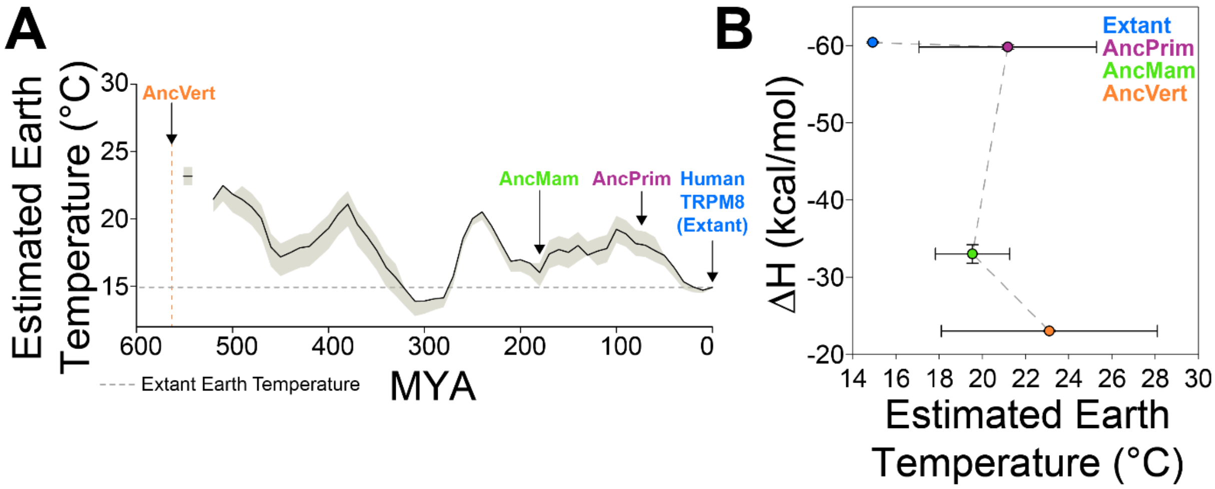

Fig. S8. Estimated earth temperatures do not correlate with the evolution of TRPM8 cold sensitivity. (**A**) Earth temperatures from the Phanerozoic eon (~540 MYA to present) were estimated by oxygen isotope measurements from shallow sea marine macroinvertebrate organisms (*56*, *57*). Estimates for earth temperature for AncVert TRPM8 (~560 MYA) were estimated from shallow sea temperatures obtained from halite inclusion analysis from ~550 MYA (*58*). (**B**) Extant and ancestral node TRPM8 cold sensitivity from Figure 3 plotted against estimated ancient and extant shallow earth sea temperatures show no obvious correlation indicative that TRPM8 thermosensitivity was driven by distinct other selection pressure(s).

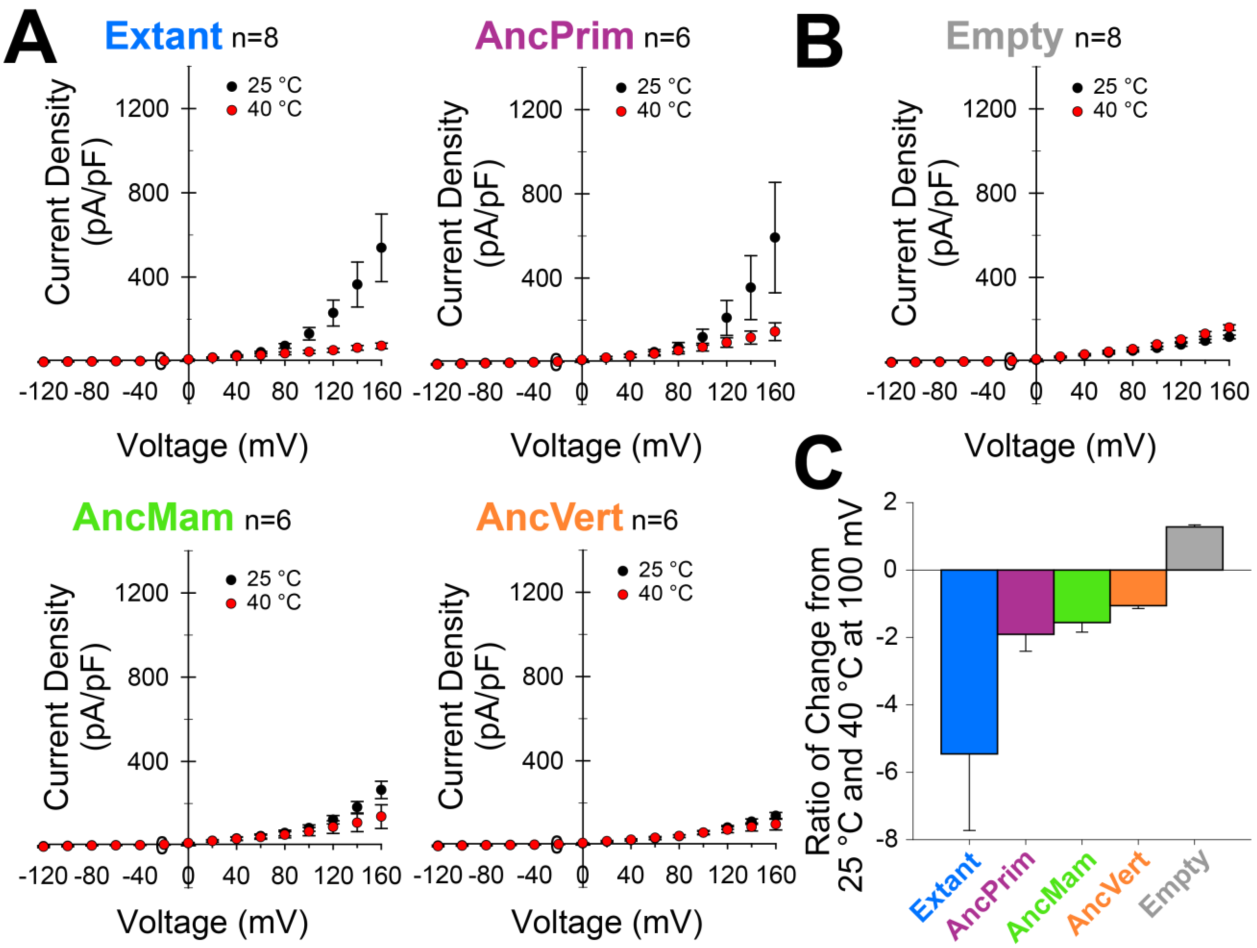

Fig. S9. Ancestral TRPM8 orthologs are not heat sensors. (**A**) Temperature-dependent whole-cell patch-clamp electrophysiology measurements show extant and ancestral TRPM8 orthologs close with heat. Temperature recorded at 25 °C and 40 °C. The data are the average of 8, 6, 6, and 6 biological replicates for Extant (blue), AncPrim (purple), AncMam (green), and AncVert (orange), respectively. (**B**) Control with the Empty pIRES2 vector (gray). (**C**) Ratio of change between 25 °C and 40 °C. Errors are in standard error of the mean.

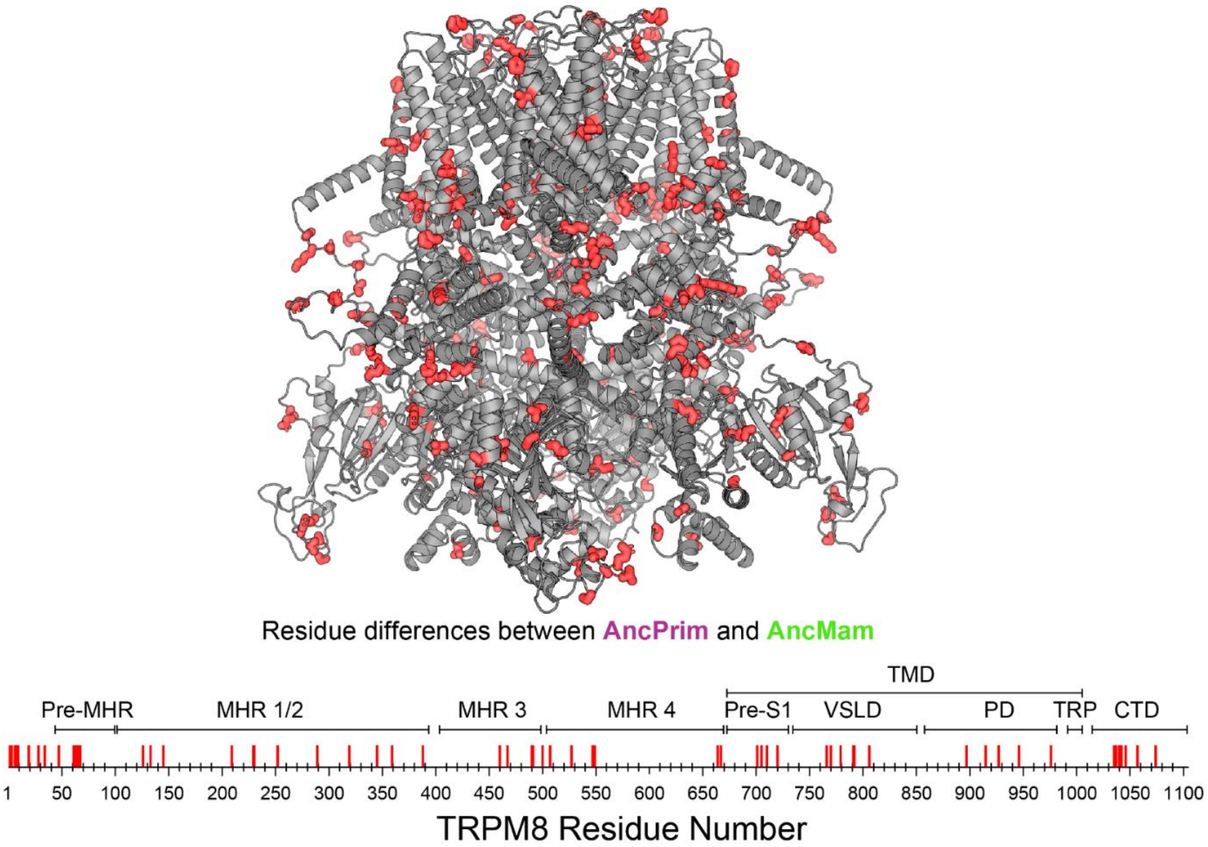

Fig. S10. Specific amino acid differences between AncPrim and AncMam. AlphaFold2 model of full-length human TRPM8 was used to highlight the amino acid differences (shown as red sticks) between the AncPrim and AncMam. A further breakdown of the amino acid differences is shown below, highlighting different domains and regions. While the distal N- and C-termini are enriched in differences between AncPrim and AncMam, the amino acid differences are widespread.

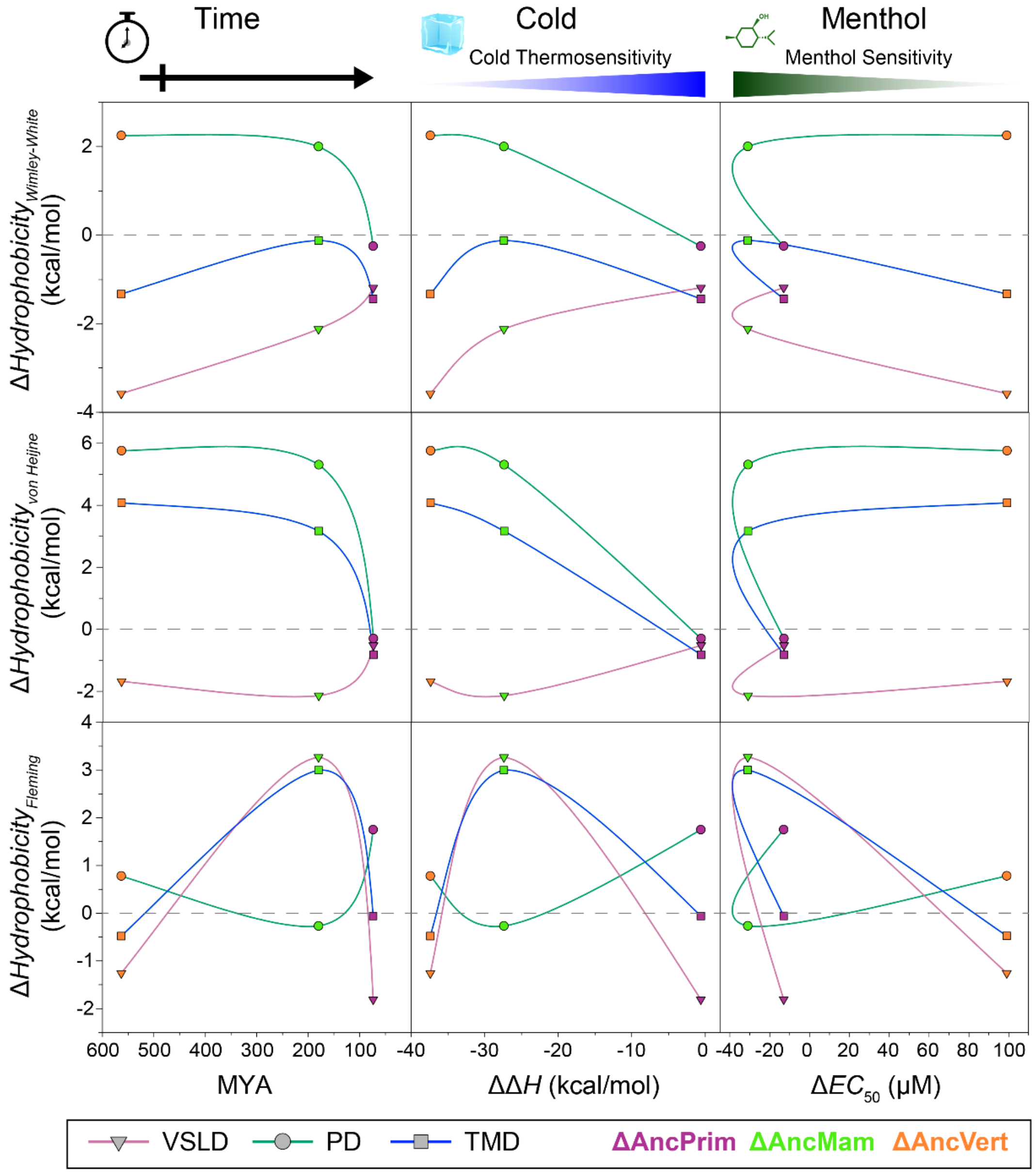

Fig. S11. Evaluation of evolutionary changes in side chain hydrophobicity of the TRPM8 transmembrane domains. Changes in side chain hydrophobicity (ΔHydrophobicity) of the S1-S4 voltage sensing-like domain (VSLD, purple lines), the S5-S6-TRP helix pore domain (PD, green lines), and the combined S1-TRP helix transmembrane domains (TMD, blue lines) for each ancestral ortholog relative to extant human TRPM8 (ΔAncPrim, ΔAncMam, and ΔAncVert respectively). This analysis was done for three independent hydrophobicity scales, Wimley-White (*64*, *127*), von Heijne (*66*), and Fleming (*65*) as top, middle, and bottom rows, respectively. The ΔHydrophobicity was plotted against evolutionary time (MYA, first column) and experimentally measured changes in TRPM8 cold sensitivity ($\Delta\Delta H$, middle column) and changes in menthol sensitivity (ΔEC_{50}).

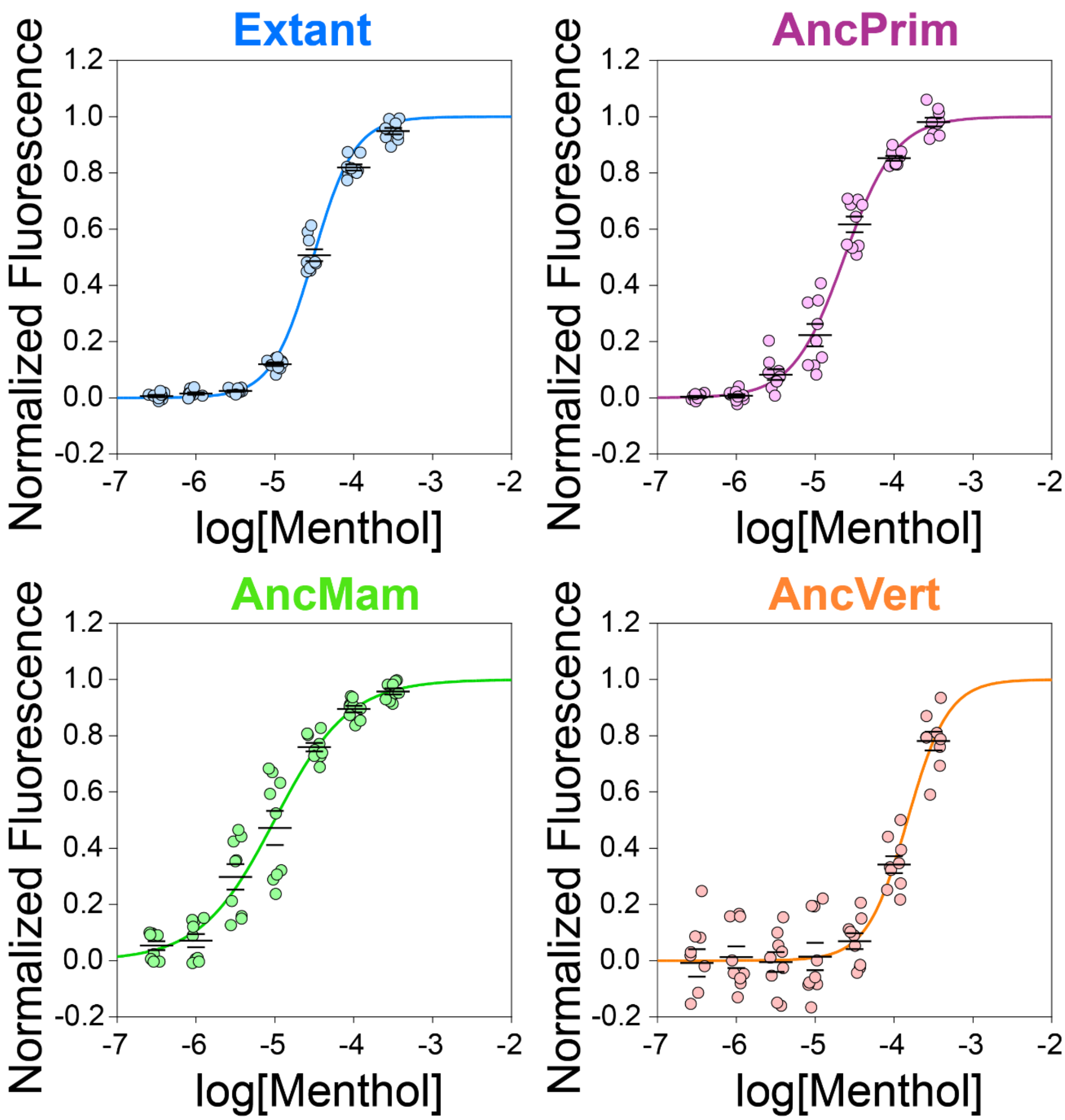

Fig. S12. Calcium mobilization assay of extant and ancestral TRPM8 orthologs. Calcium mobilization assay curves with mean (middle band) and SEM (top and bottom band) at each temperature with jitters of each experiment. Biological replicates of 9 were done for extant and TRPM8 orthologs. AncVert menthol response were weak towards the lower concentration resulting in higher variance.

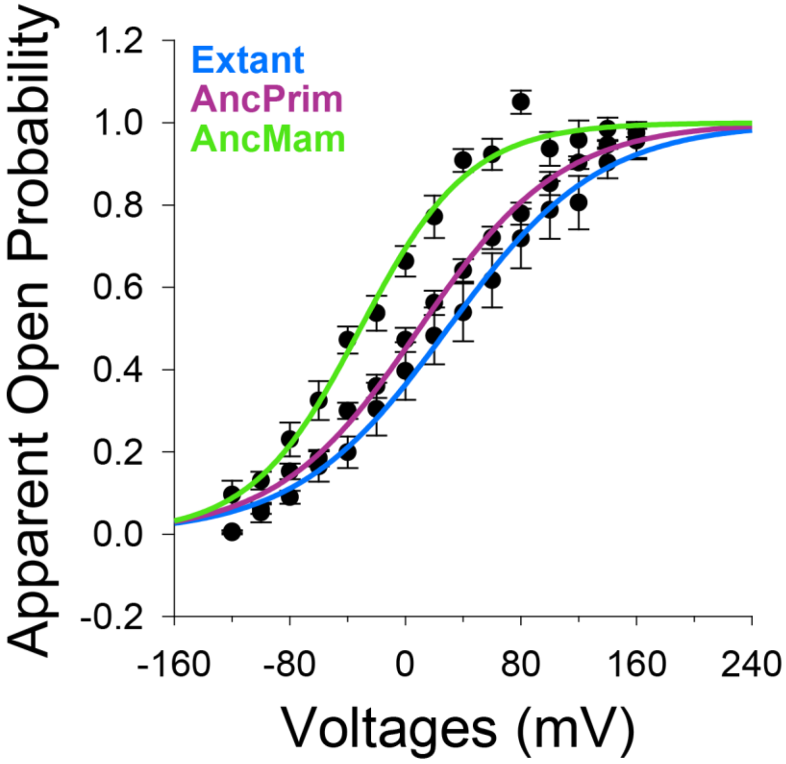

Fig. S13. Electrophysiology-base evaluation of voltage-menthol coupling from tail current measurements. The voltage-menthol coupling from tail currents at 310 ms in the presence of 500 µM menthol. The data was scaled to the upper and lower bonds and from averages of 8, 9, 11, and 8 different cells for Extant (blue), AncPrim (purple), AncMam (green), and AncVert (orange), respectively. Errors are in standard error of the mean. AncVert data were not included because the voltage-menthol coupling was substantially right shifted that it didn't show two state behavior.

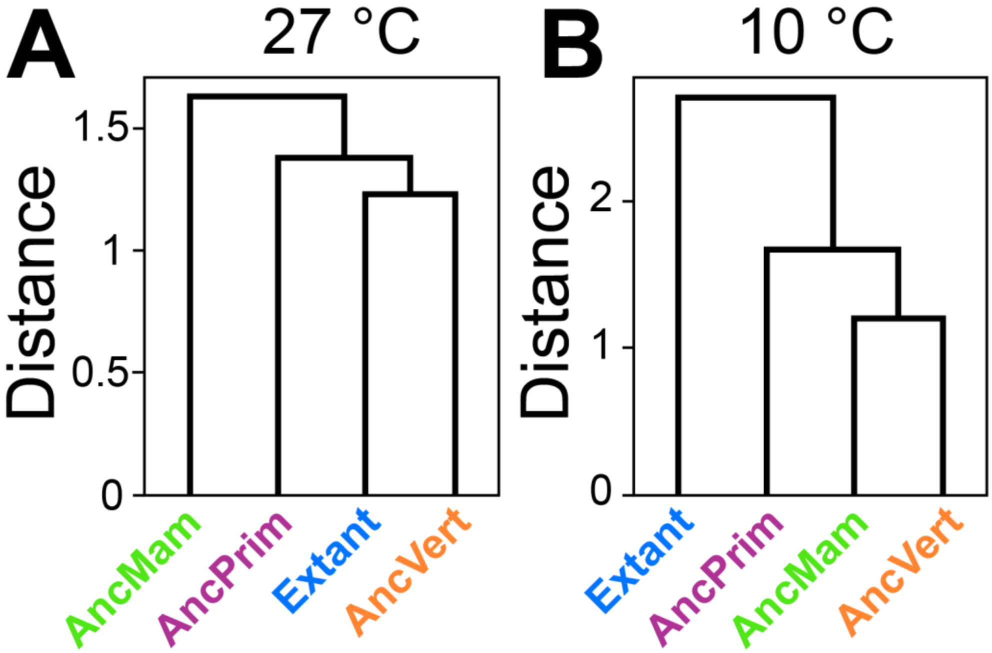

Fig. S14. Dynamic Flexibility Index (DFI) clustering recapitulates profiles of ancestral and extant human TRPM8 at 27 °C and 10 °C. Dendrograms were constructed using the top three modes obtained from the principal component analysis of the DFI profiles. The distance refers to the pairwise distances between the extant and the ancestral variants in the lower dimensional principal component space. (**A**) Comparison of the two dendrograms reveals that the dynamics profiles obtained using the MD trajectories at 27 °C recapitulates the order of extant and ancestral TRPM8 menthol sensitivity. (**B**) Analogous DFI analysis from cold temperature simulations (10 °C) captures the evolution of the cold sensing, clustering the lower temperature-sensing AncMam and AncVert TRPM8 together. AncPrim is in the same branch but still significantly different than the more ancient and less temperature-sensitive AncMam and AncVert ancestors.

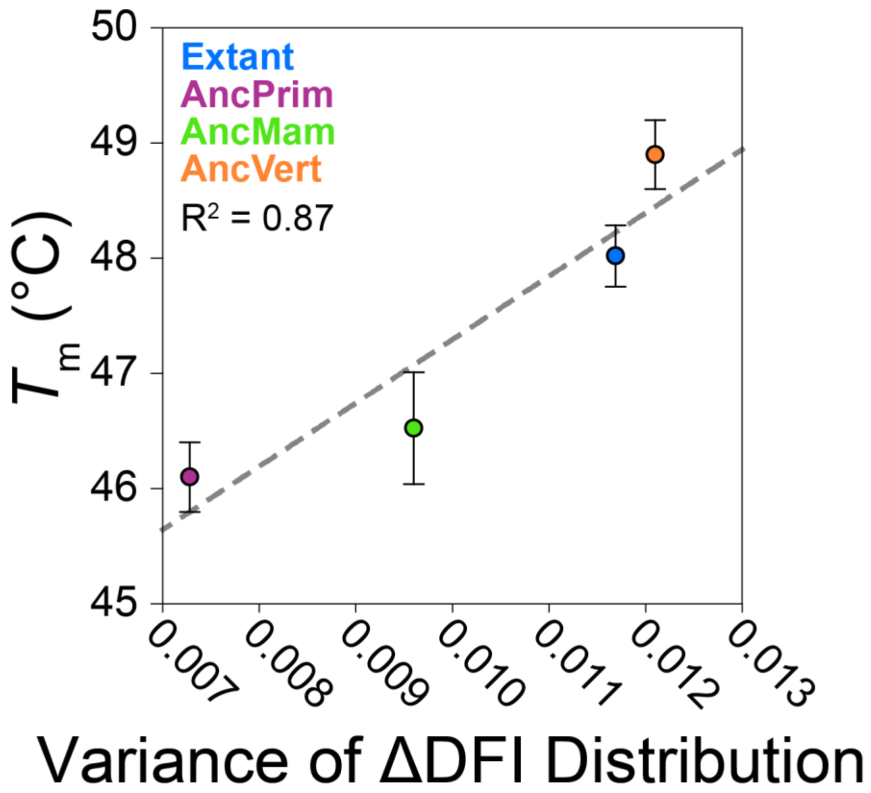

Fig. S15. Temperature-dependent differences in calculated flexibility against cellular stability. A linear relationship with an R^2 value of 0.87 is seen with the T_m and variance of ΔDFI distribution shows that computational analysis correlates with the experimental CETSA results.

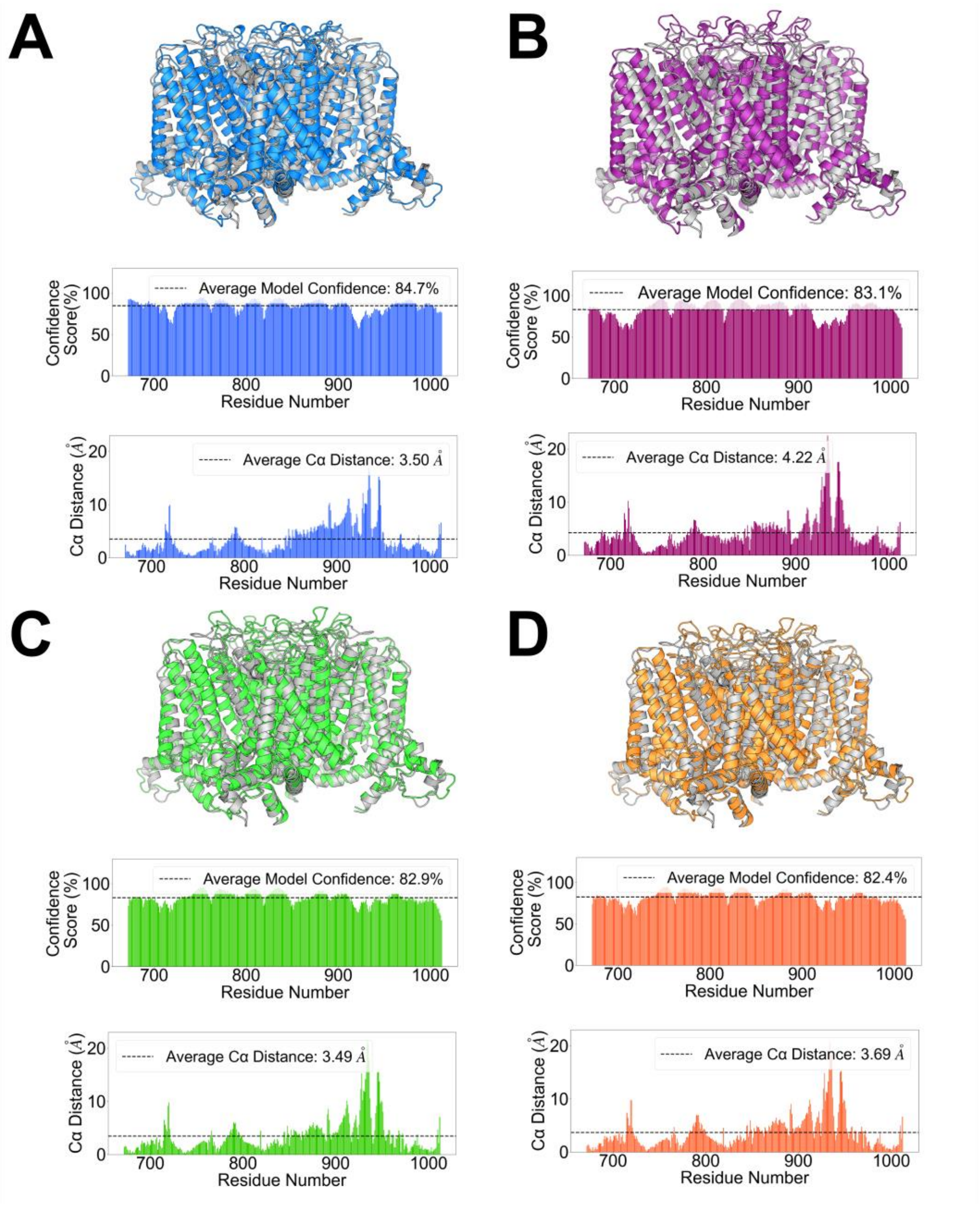

Fig. S16. Comparison of RosettaCM modeled structures with those obtained from AlphaFold. The AlphaFold and the RosettaCM structures superimposed on each other along with the confidence score plots and the per residue C$\boldsymbol{\alpha}$ distances between the structures modeled using the two approaches for the (**A**) Extant, (**B**) AncPrim, (**C**) AncMam and (**D**) AncVert respectively. The AlphaFold structures are shown in color (blue for Extant, purple for AncPrim, green for AncMam and orange for AncVert) whereas the Rosetta overlays are in gray. While the total Root Mean Square Deviation (RMSD) are shown as legends in the distance plots, the total of RMSDs, computed over only helices, between the two modelled structures per protein follows as 2.7 Å for Extant, 3.27 Å for AncPrim, 2.59 Å for AncMam and 2.82 Å for AncVert.

Table S1. Per domain analysis of the number and percentage of residue variance in extant and ancestral TRPM8 orthologs.

Ortholog	Full-Length (# of residues [%])	N-terminus (# of residues [%])	Transmembrane (# of residues [%])	C-terminus (# of residues [%])
**Extant**	1104 [100%]	671 [61%]	341 [31%]	91 [8%]
**AncPrim**	1104 [100%]	671 [61%]	341 [31%]	91 [8%]
**AncMam**	1101 [100%]	668 [61%]	341 [31%]	91 [8%]
**AncVert**	1093 [100%]	660 [60%]	341 [31%]	91 [8%]

Table S2. Amino acid variance of the ancestral TRPM8 orthologs relative to extant human TRPM8.

Ortholog	ΔFull- Length (# of residues)	ΔN-terminus (# of residues)	ΔTransmembrane (# of residues)	ΔC-terminus (# of residues)
**AncPrim**	23	17	4	2
**AncMam**	86	57	19	10
**AncVert**	124	80	29	15

Table S3. The percentage of amino acid differences between the extant and each of the ancestral orthologs in each domain.

Ortholog	Full- Length (% [Fraction])	ΔN-terminus (% [Fraction])	ΔTransmembrane (% [Fraction])	ΔC-terminus (% [Fraction])
**AncPrim**	2.1% [23/1104]	2.5% [17/671]	1.2% [4/341]	2.2% [2/91]
**AncMam**	8.8% [86/1101]	8.5% [57/671]	5.6% [19/341]	11.0% [10/91]
**AncVert**	11.3% [124/1093]	11.9% [80/671]	8.5% [29/341]	16.5% [15/91]

Table S4. Statistical significance determined from unpaired t-test

Flow Cytometry	Extant	AncPrim	AncMam	AncVert	Control
**Extant**	—	ns	ns	ns	—
**AncPrim**	0.9416	—	ns	ns	—
**AncMam**	0.2042	0.198	—	ns	—
**AncVert**	0.4014	0.4087	0.6782	—	—
**Control**	—	—	—	—	—

CETSA	Extant	AncPri	AncMam	AncVert	Control
**Extant**	—	**	*	ns	—
**AncPrim**	0.0021	—	ns	***	—
**AncMam**	0.0257	0.4825	—	**	—
**AncVert**	0.0608	0.0003	0.0035	—	—
**Control**	—	—	—	—	—

Cold Enthalpy	Extant	AncPrim	AncMam	AncVert	Control
**Extant**	—	****	****	****	****
**AncPrim**	<0.0001	—	****	****	****
**AncMam**	<0.0001	<0.0001	—	****	****
**AncVert**	<0.0001	<0.0001	<0.0001	—	****
**Control**	<0.0001	<0.0001	<0.0001	<0.0001	—

Normalized Menthol Response	Extant	AncPrim	AncMam	AncVert	Control
**Extant**	—	ns	ns	***	**
**AncPrim**	0.4542	—	ns	***	**
**AncMam**	0.1361	0.5453	—	***	***
**AncVert**	0.0006	0.0006	0.0001	—	ns
**Control**	0.0022	0.0056	0.0007	0.097	—

Menthol EC_{50}	Extant	AncPrim	AncMam	AncVert	Control
**Extant**	—	****	****	****	—
**AncPrim**	<0.0001	—	****	****	—
**AncMam**	<0.0001	<0.0001	—	****	—
**AncVert**	<0.0001	<0.0001	<0.0001	—	—
**Control**	—	—	—	—	—

Table S5. Hydrophobicity scale values used to evaluate side chain hydrophobicity

Residue	*Wimley-White* (*64, 127*) (kcal/mol)	*von Heijne* (*66*) (kcal/mol)	*Fleming* (*65*) (kcal/mol)
A	0.11	0.11	0
C	0.22	-0.13	0.49
D	2.41	3.49	2.95
E	0.12	2.68	1.64
F	0.58	-0.32	-2.2
G	1.14	0.74	1.72
H	-0.06	2.06	4.76
I	-0.81	-0.6	-1.56
K	1.81	2.71	5.39
L	-0.69	-0.55	-1.81
M	-0.44	-0.1	-0.76
N	0.43	2.05	3.47
P	-0.31	2.23	-1.52
Q	0.19	2.36	3.01
R	1	2.58	3.71
S	0.33	0.84	1.83
T	0.11	0.52	1.78
V	0.53	-0.31	-0.78
W	-0.24	0.3	-0.38
Y	0.23	0.68	-1.09

Table S6. Evaluation of full-length and subdomains of the extant and ancestral human TRPM8 hydrophobicity with the biological hydrophobicity scale.[a]

		Extant hTRPM8[b]	AncPrim	AncMam	AncVert	
**Full Length**	$\sum \Delta G_{BiolHydro}$	1062.9	1067.1	1081.8	1071.2	*kcal/mol*
	$\Delta\Delta G_{BiolHydro}$	*n/a*	4.2	18.9	8.3	*kcal/mol*
**N-terminus**	$\sum \Delta G_{BiolHydro}$	764.5	769.5	776.4	768.7	*kcal/mol*
	$\Delta\Delta G_{BiolHydro}$	*n/a*	5.0	11.9	4.3	*kcal/mol*
**C-terminus**	$\sum \Delta G_{BiolHydro}$	104.5	104.5	108.3	105.6	*kcal/mol*
	$\Delta\Delta G_{BiolHydro}$	*n/a*	0.1	3.8	1.2	*kcal/mol*
**Soluble Termini (N&C-term)**	$\sum \Delta G_{BiolHydro}$	868.9	874.0	884.7	874.4	*kcal/mol*
	$\Delta\Delta G_{BiolHydro}$	*n/a*	5.1	15.8	5.4	*kcal/mol*
**PreS1 & TMD & TRP Helix**	$\sum \Delta G_{BiolHydro}$	240.8	239.7	241.6	242.8	*kcal/mol*
	$\Delta\Delta G_{BiolHydro}$	*n/a*	-1.1	0.8	1.9	*kcal/mol*
**VSLD (S1-S4)**	$\sum \Delta G_{BiolHydro}$	63.1	62.6	61.0	61.4	*kcal/mol*
	$\Delta\Delta G_{BiolHydro}$	*n/a*	-0.5	-2.1	-1.7	*kcal/mol*
**PD (S5-S6)**	$\sum \Delta G_{BiolHydro}$	88.1	87.8	93.4	93.8	*kcal/mol*
	$\Delta\Delta G_{BiolHydro}$	*n/a*	-0.3	5.3	5.8	*kcal/mol*
**TMD (S1-S6)**	$\sum \Delta G_{BiolHydro}$	158.3	157.5	161.5	162.4	*kcal/mol*
	$\Delta\Delta G_{BiolHydro}$	*n/a*	-0.8	3.2	4.1	*kcal/mol*

[a] Values based on Hessa et al. (*66*).
[b] Definition of the domains: Full length: 1-1104; N-terminus: 1-731; C-terminus: 1014-1104 (91 residues); PreS1/TMD/TRP helix: 672-1006 (335 residues); VSLD: 734-851 (118 residues); PD: 857-982 (126 residues); TMD: 734-982 (249 residues). *n.b.* that some stretches include loops; for example, the TMD includes loop residues between the VSLD and PD, which is why the sum of the VSLD and PD are not equivalent to the TMD.

Table S7. DNA oligo primers used in these studies.

Primer Name	Sequence	Use
pKanAttB-2M8-F	aatcaaataaagcatgcgggatccaccg	Amplify pKanAttB vector
pKanAttB-2M8-R	gaaaggacattggtggcggatgatcctg	Amplify pKanAttB vector
hM8LLP-Int-F	tccgccaccaatgtcctttcgggcagcc	Amplify extant human TRPM8 gene
hM8LLP-Int-R	cccgcatgctttatttgattttattagcaatctctttcagaagacc	Amplify extant human TRPM8 gene
pKanAttB-2AncP-F	gattaagtgaagcatgcgggatccaccg	Amplify pKanAttB vector
pKanAttB-2AncP-R	cgaaagacattggtggcggatgatcctg	Amplify pKanAttB vector
AncPrim-LLP-Int-F	tccgccaccaatgtctttcgagggcgctag	Amplify AncPrim TRPM8 gene
AncPrim-LLP-Int-R	cccgcatgcttcacttaatcttgttagcgatctcc	Amplify AncPrim TRPM8 gene
pKanAttB-2AncM-F	gattaagtaaagcatgcgggatccaccg	Amplify pKanAttB vector
pKanAttB-2AncM-R	ggaagtgcattggtggcggatgatcctg	Amplify pKanAttB vector
AncMam-LLP-Int-F	tccgccaccaatgcacttccagtccgctatg	Amplify AncMam TRPM8 gene
AncMam-LLP-Int-R	cccgcatgctttacttaatcttgttagcgatctccttg	Amplify AncMam TRPM8 gene
pKanAttB-2AncV-F	gattaagtaaagcatgcgggatccaccg	Amplify pKanAttB vector
pKanAttB-2AncV-R	ggttgcgcattggtggcggatgatcctg	Amplify pKanAttB vector
AncVert-LLP-Int-F	tccgccaccaatgcgcaaccggcgcaac	Amplify AncVert TRPM8 gene
AncVert-LLP-Int-R	cccgcatgctttacttaatcttgttagcgatctccttgaggagtc	Amplify AncVert TRPM8 gene

Data S1. Multiple sequence alignment of 32 TRPM8 species to accurately determine divergence times.

Data S2. Multiple sequence alignment of 21 TRPM8 species selected to investigate the molecular evolution and elucidate selection pressure on critical regions.

Data S3. Multiple sequence alignment of a diverse collection of 250 TRPM8 species.

Data S4. Source data are provided for Fig. 2, 3, 4, 6, S5, S6, S7, S8, S9, S11, S12, S13 and S15.

REFERENCES AND NOTES

1. D. D. McKemy, W. M. Neuhausser, D. Julius, Identification of a cold receptor reveals a general role for TRP channels in thermosensation. *Nature* **416**, 52–58 (2002).

2. A. M. Peier, A. Moqrich, A. C. Hergarden, A. J. Reeve, D. A. Andersson, G. M. Story, T. J. Earley, I. Dragoni, P. McIntyre, S. Bevan, A. Patapoutian, A TRP channel that senses cold stimuli and menthol. *Cell* **108**, 705–715 (2002).

3. H. Hensel, Y. Zotterman, The effect of menthol on the thermoreceptors. *Acta Physiol. Scand.* **24**, 27–34 (1951).

4. W. J. Winchester, K. Gore, S. Glatt, W. Petit, J. C. Gardiner, K. Conlon, M. Postlethwaite, P. P. Saintot, S. Roberts, J. R. Gosset, T. Matsuura, M. D. Andrews, P. A. Glossop, M. J. Palmer, N. Clear, S. Collins, K. Beaumont, D. S. Reynolds, Inhibition of TRPM8 channels reduces pain in the cold pressor test in humans. *J Pharmacol Exp Ther.* **351**, 259–269 (2014).

5. D. B. Horne, K. Biswas, J. Brown, M. D. Bartberger, J. Clarine, C. D. Davis, V. K. Gore, S. Harried, M. Horner, M. R. Kaller, S. G. Lehto, Q. Liu, V. V. Ma, H. Monenschein, T. T. Nguyen, C. C. Yuan, B. D. Youngblood, M. Zhang, W. Zhong, J. R. Allen, J. J. Chen, N. R. Gavva, Discovery of TRPM8 Antagonist (S)-6-(((3-fluoro-4-(trifluoromethoxy)phenyl)(3-fluoropyridin-2-yl)methyl)carbamoyl)nicotinic acid (AMG 333), a clinical candidate for the treatment of migraine. *J. Med. Chem.* **61**, 8186–8201 (2018).

6. C. Izquierdo, M. Martín-Martínez, I. Gómez-Monterrey, R. González-Muñiz, TRPM8 channels: Advances in structural studies and pharmacological modulation. *Int. J. Mol. Sci.* **22**, 8502 (2021).

7. S. Brauchi, P. Orio, R. Latorre, Clues to understanding cold sensation: Thermodynamics and electrophysiological analysis of the cold receptor TRPM8. *Proc. Natl. Acad. Sci. U.S.A.* **101**, 15494–15499 (2004).

8. R. Latorre, S. Brauchi, G. Orta, C. Zaelzer, G. Vargas, ThermoTRP channels as modular proteins with allosteric gating. *Cell Calcium* **42**, 427–438 (2007).

9. J. A. Matta, G. P. Ahern, Voltage is a partial activator of rat thermosensitive TRP channels. *Physiol. J.* **585**, 469–482 (2007).

10. T. Voets, G. Droogmans, U. Wissenbach, A. Janssens, V. Flockerzi, B. Nilius, The principle of temperature-dependent gating in cold- and heat-sensitive TRP channels. *Nature* **430**, 748–754 (2004).

11. J. E. Prinston, J. R. Emlaw, M. F. Dextraze, C. J. G. Tessier, F. J. Pérez-Areales, M. S. McNulty, C. J. B. daCosta, Ancestral reconstruction approach to acetylcholine receptor structure and function. *Structure* **25**, 1295–1302.e3 (2017).

12. C. J. G. Tessier, R. M. Sturgeon, J. R. Emlaw, G. D. McCluskey, F. J. Pérez-Areales, C. J. B. daCosta, Ancestral acetylcholine receptor β-subunit forms homopentamers that prime before opening spontaneously. *eLife* **11**, e76504 (2022).

13. S. Saito, R. Shingai, Evolution of thermoTRP ion channel homologs in vertebrates. *Physiol. Genomics* **27**, 219–230 (2006).

14. S. Ma, H. Yu, Z. Zhao, Z. Luo, J. Chen, Y. Ni, R. Jin, L. Ma, P. Wang, Z. Zhu, L. Li, J. Zhong, D. Liu, B. Nilius, Z. Zhu, Activation of the cold-sensing TRPM8 channel triggers UCP1-dependent thermogenesis and prevents obesity. *J. Mol. Cell Biol.* **4**, 88–96 (2012).

15. R. Araújo, R. David, J. Benoit, J. K. Lungmus, A. Stoessel, P. M. Barrett, J. A. Maisano, E. Ekdale, M. Orliac, Z.-X. Luo, A. G. Martinelli, E. A. Hoffman, C. A. Sidor, R. M. S. Martins, F. Spoor, K. D. Angielczyk, Inner ear biomechanics reveals a Late Triassic origin for mammalian endothermy. *Nature* **607**, 726–731 (2022).

16. P. McIntyre, L. M. McLatchie, A. Chambers, E. Phillips, M. Clarke, J. Savidge, C. Toms, M. Peacock, K. Shah, J. Winter, N. Weerasakera, M. Webb, H. P. Rang, S. Bevan, I. F. James, Pharmacological differences between the human and rat vanilloid receptor 1 (VR1). *Br. J. Pharmacol.* **132**, 1084–1094 (2001).

17. A. Garami, Y. P. Shimansky, Z. Rumbus, R. C. L. Vizin, N. Farkas, J. Hegyi, Z. Szakacs, M. Solymar, A. Csenkey, D. A. Chiche, R. Kapil, D. J. Kyle, W. D. Van Horn, P. Hegyi, A. A. Romanovsky,

Hyperthermia induced by transient receptor potential vanilloid-1 (TRPV1) antagonists in human clinical trials: Insights from mathematical modeling and meta-analysis. *Pharmacol. Ther.* **208**, 107474 (2020).

18. J. K. Hilton, T. Salehpour, N. J. Sisco, P. Rath, W. D. Van Horn, Phosphoinositide-interacting regulator of TRP (PIRT) has opposing effects on human and mouse TRPM8 ion channels. *J. Biol. Chem.* **293**, 9423–9434 (2018).

19. V. B. Journigan, D. Alarcón-Alarcón, Z. Feng, Y. Wang, T. Liang, D. C. Dawley, A. Amin, C. Montano, W. D. Van Horn, X. Q. Xie, A. Ferrer-Montiel, A. Fernández-Carvajal, Structural and in vitro functional characterization of a menthyl trpm8 antagonist indicates species-dependent regulation. *ACS Med. Chem. Lett.* **12**, 758–767 (2021).

20. A. I. Caceres, B. Liu, S. V. Jabba, S. Achanta, J. B. Morris, S. E. Jordt, Transient receptor potential cation channel subfamily m member 8 channels mediate the anti-inflammatory effects of eucalyptol. *Br. J. Pharmacol.* **174**, 867–879 (2017).

21. C. J. Gordon, The mouse thermoregulatory system: Its impact on translating biomedical data to humans. *Physiol. Behav.* **179**, 55–66 (2017).

22. V. Škop, J. Guo, N. Liu, C. Xiao, K. D. Hall, O. Gavrilova, M. L. Reitman, Mouse thermoregulation: Introducing the concept of the thermoneutral point. *Cell Rep.* **31**, 107501 (2020).

23. R. J. Seeley, O. A. MacDougald, Mice as experimental models for human physiology: When several degrees in housing temperature matter. *Nat. Metab.* **3**, 443–445 (2021).

24. S. F. Morrison, K. Nakamura, D. Tupone, Thermoregulation in mice: The road to understanding torpor hypothermia and the shortcomings of a circuit for generating fever. *Temperature* **9**, 8–11 (2022).

25. S. Kumar, M. Sanderford, V. E. Gray, J. Ye, L. Liu, Evolutionary diagnosis method for variants in personal exomes. *Nat. Methods* **9**, 855–856 (2012).

26. J. K. Hilton, M. Kim, W. D. Van Horn, Structural and evolutionary insights point to allosteric regulation of TRP ion channels. *Acc. Chem. Res.* **52**, 1643–1652 (2019).

27. M. Bandell, A. E. Dubin, M. J. Petrus, A. Orth, J. Mathur, S. W. Hwang, A. Patapoutian, High-throughput random mutagenesis screen reveals TRPM8 residues specifically required for activation by menthol. *Nat. Neurosci.* **9**, 493–500 (2006).

28. A. Malkia, M. Pertusa, G. Fernández-Ballester, A. Ferrer-Montiel, F. Viana, Differential role of the menthol-binding residue Y745 in the antagonism of thermally gated TRPM8 channels. *Mol. Pain* **5**, 62 (2009).

29. T. Voets, G. Owsianik, A. Janssens, K. Talavera, B. Nilius, TRPM8 voltage sensor mutants reveal a mechanism for integrating thermal and chemical stimuli. *Nat. Chem. Biol.* **3**, 174–182 (2007).

30. A. Janssens, T. Voets, Ligand stoichiometry of the cold- and menthol-activated channel TRPM8. *J. Physiol.* **589**, 4827–4835 (2011).

31. P. Rath, J. K. Hilton, N. J. Sisco, W. D. Van Horn, Implications of human transient receptor potential melastatin 8 (TRPM8) channel gating from menthol binding studies of the sensing domain. *Biochemistry* **55**, 114–124 (2016).

32. Y. Yin, S. C. Le, A. L. Hsu, M. J. Borgnia, H. Yang, S.-Y. Lee, Structural basis of cooling agent and lipid sensing by the cold-activated TRPM8 channel. *Science* **363**, eaav9334 (2019).

33. M. M. Diver, Y. Cheng, D. Julius, Structural insights into TRPM8 inhibition and desensitization. *Science* **365**, 1434–1440 (2019).

34. C. Zhao, Y. Xie, L. Xu, F. Ye, X. Xu, W. Yang, F. Yang, J. Guo, Structures of a mammalian TRPM8 in closed state. *Nat. Commun.* **13**, 3113 (2022).

35. N. J. Sisco, C. V. M. Helsell, W. D. Van Horn, Competitive interactions between PIRT, the cold sensing ion channel TRPM8, and PIP_2 suggest a mechanism for regulation. *Sci. Rep.* **9**, 14128 (2019).

36. T. Rohács, C. M. B. Lopes, I. Michailidis, D. E. Logothetis, PI(4,5)P2 regulates the activation and desensitization of TRPM8 channels through the TRP domain. *Nat. Neurosci.* **8**, 626–634 (2005).

37. Y. Yin, M. Wu, L. Zubcevic, W. F. Borschel, G. C. Lander, S. Y. Lee, Structure of the cold- and menthol-sensing ion channel TRPM8. *Science* **359**, 237–241 (2018).

38. Y. Yin, F. Zhang, S. Feng, K. J. Butay, M. J. Borgnia, W. Im, S.-Y. Lee, Activation mechanism of the mouse cold-sensing TRPM8 channel by cooling agonist and PIP_2. *Science* **378**, eadd1268 (2022).

39. H.-H. Chuang, W. M. Neuhausser, D. Julius, The super-cooling agent icilin reveals a mechanism of coincidence detection by a temperature-sensitive TRP channel. *Neuron* **43**, 859–869 (2004).

40. A. Pedretti, C. Marconi, I. Bettinelli, G. Vistoli, Comparative modeling of the quaternary structure for the human TRPM8 channel and analysis of its binding features. *Biochim. Biophys. Acta* **1788**, 973–982 (2009).

41. B. R. Myers, Y. M. Sigal, D. Julius, Evolution of thermal response properties in a cold-activated TRP channel. *PLOS ONE* **4**, e5741 (2009).

42. L. Tsavaler, M. H. Shapero, S. Morkowski, R. Laus, Trp-p8, a novel prostate-specific gene, is up-regulated in prostate cancer and other malignancies and shares high homology with transient receptor potential calcium channel proteins. *Cancer Res.* **61**, 3760–3769 (2001).

43. C. J. Proudfoot, E. M. Garry, D. F. Cottrell, R. Rosie, H. Anderson, D. C. Robertson, S. M. Fleetwood-Walker, R. Mitchell, Analgesia mediated by the TRPM8 cold receptor in chronic neuropathic pain. *Curr. Biol.* **16**, 1591–1605 (2006).

44. L. M. Chamness, N. B. Zelt, H. R. Harrington, C. P. Kuntz, B. J. Bender, W. D. Penn, J. J. Ziarek, J. Meiler, J. P. Schlebach, Molecular basis for the evolved instability of a human G-protein coupled receptor. *Cell Rep.* **37**, 110046 (2021).

45. J. T. Marinko, B. D. Carter, C. R. Sanders, Direct relationship between increased expression and mistrafficking of the charcot-marie-tooth-associated protein PMP22. *J. Biol. Chem.* **295**, 11963–11970 (2020).

46. H. Huang, L. M. Chamness, C. G. Vanoye, G. Kuenze, J. Meiler, A. L. George, J. P. Schlebach, C. R. Sanders, Disease-linked supertrafficking of a potassium channel. *J. Biol. Chem.* **296**, 1–14 (2021).

47. D. M. Molina, R. Jafari, M. Ignatushchenko, T. Seki, E. A. Larsson, C. Dan, L. Sreekumar, Y. Cao, P. Nordlund, Monitoring drug target engagement in cells and tissues using the cellular thermal shift assay. *Science* **341**, 84–87 (2013).

48. A. Kawatkar, M. Schefter, N. O. Hermansson, A. Snijder, N. Dekker, D. G. Brown, T. Lundbäck, A. X. Zhang, M. P. Castaldi, CETSA beyond soluble targets: A broad application to multipass transmembrane proteins. *ACS Chem. Biol.* **14**, 1913–1920 (2019).

49. R. Jafari, H. Almqvist, H. Axelsson, M. Ignatushchenko, T. Lundbäck, P. Nordlund, D. M. Molina, The cellular thermal shift assay for evaluating drug target interactions in cells. *Nat. Protoc.* **9**, 2100–2122 (2014).

50. M. Kim, N. J. Sisco, J. K. Hilton, C. M. Montano, M. A. Castro, B. R. Cherry, M. Levitus, W. D. Van Horn, Evidence that the TRPV1 S1-S4 membrane domain contributes to thermosensing. *Nat. Commun.* **11**, 4169 (2020).

51. D. D. Luu, A. M. Owens, M. D. Mebrat, W. D. Van Horn, A molecular perspective on identifying TRPV1 thermosensitive regions and disentangling polymodal activation. *Temperature* **10**, 67–10 (2021).

52. T. Voets, TRP channels and thermosensation. *Handb. Exp. Pharmacol.* **223**, 729–741 (2014).

53. D. E. Clapham, C. Miller, A thermodynamic framework for understanding temperature sensing by transient receptor potential (TRP) channels. *Proc. Natl. Acad. Sci. U.S.A.* **108**, 19492–19497 (2011).

54. V. Matos-Cruz, E. R. Schneider, M. Mastrotto, D. K. Merriman, S. N. Bagriantsev, E. O. Gracheva, Molecular prerequisites for diminished cold sensitivity in ground squirrels and hamsters. *Cell Rep.* **21**, 3329–3337 (2017).

55. V. Nguyen, C. Wilson, M. Hoemberger, J. B. Stiller, R. V. Agafonov, S. Kutter, J. English, D. L. Theobald, D. Kern, Evolutionary drivers of thermoadaptation in enzyme catalysis. *Science* **355**, 289–294 (2017).

56. J. Veizer, D. Ala, K. Azmy, P. Bruckschen, D. Buhl, F. Bruhn, G. A. F. Carden, A. Diener, S. Ebneth, Y. Godderis, T. Jasper, C. Korte, F. Pawellek, O. G. Podlaha, H. Strauss, ^{87}Sr/^{86}Sr, δ^{13}C and δ^{18}O evolution of Phanerozoic seawater. *Chem. Geol.* **161**, 59–88 (1999).

57. J. C. Zachos, G. R. Dickens, R. E. Zeebe, An early Cenozoic perspective on greenhouse warming and carbon-cycle dynamics. *Nature* **451**, 279–283 (2008).

58. F. Meng, P. Ni, J. D. Schiffbauer, X. Yuan, C. Zhou, Y. Wang, M. Xia, Ediacaran seawater temperature: Evidence from inclusions of Sinian halite. *Precambrian Res.* **184**, 63–69 (2011).

59. I. Vandewauw, K. De Clercq, M. Mulier, K. Held, S. Pinto, N. Van Ranst, A. Segal, T. Voet, R. Vennekens, K. Zimmermann, J. Vriens, T. Voets, A TRP channel trio mediates acute noxious heat sensing. *Nature* **555**, 662–666 (2018).

60. J. Chen, D. Kang, J. Xu, M. Lake, J. O. Hogan, C. Sun, K. Walter, B. Yao, D. Kim, Species differences and molecular determinant of TRPA1 cold sensitivity. *Nat. Commun.* **4**, 2501 (2013).

61. S. Jabba, R. Goyal, J. O. Sosa-Pagán, H. Moldenhauer, J. Wu, B. Kalmeta, M. Bandell, R. Latorre, A. Patapoutian, J. Grandl, Directionality of temperature activation in mouse TRPA1 ion channel can be inverted by single-point mutations in ankyrin repeat six. *Neuron* **82**, 1017–1031 (2014).

62. S. Yang, X. Lu, Y. Wang, L. Xu, X. Chen, F. Yang, R. Lai, A paradigm of thermal adaptation in penguins and elephants by tuning cold activation in TRPM8. *Proc. Natl. Acad. Sci. U.S.A.* **117**, 8633–8638 (2020).

63. M. Pertusa, B. Rivera, A. González, G. Ugarte, R. Madrid, Critical role of the pore domain in the cold response of TRPM8 channels identified by ortholog functional comparison. *J. Biol. Chem.* **293**, 12454–12471 (2018).

64. W. C. Wimley, S. H. White, Experimentally determined hydrophobicity scale for proteins at membrane interfaces. *Nat. Struct. Mol. Biol.* **3**, 842–848 (1996).

65. C. P. Moon, K. G. Fleming, Side-chain hydrophobicity scale derived from transmembrane protein folding into lipid bilayers. *Proc. Natl. Acad. Sci. U.S.A.* **108**, 10174–10177 (2011).

66. T. Hessa, H. Kim, K. Bihlmaier, C. Lundin, J. Boekel, H. Andersson, I. M. Nilsson, S. H. White, G. Von Heijne, Recognition of transmembrane helices by the endoplasmic reticulum translocon. *Nature* **433**, 377–381 (2005).

67. D. Colquhoun, Binding, gating, affinity and efficacy: The interpretation of structure-activity relationships for agonists and of the effects of mutating receptors. *Br. J. Pharmacol.* **125**, 923–947 (1998).

68. T. Modi, V. A. Risso, S. Martinez-Rodriguez, J. A. Gavira, M. D. Mebrat, W. D. Van Horn, J. M. Sanchez-Ruiz, S. Banu Ozkan, Hinge-shift mechanism as a protein design principle for the evolution of β-lactamases from substrate promiscuity to specificity. *Nat. Commun.* **12**, 1852 (2021).

69. T. Modi, P. Campitelli, I. C. Kazan, S. B. Ozkan, Protein folding stability and binding interactions through the lens of evolution: A dynamical perspective. *Curr. Opin. Struct. Biol.* **66**, 207–215 (2021).

70. K. E. Larrimore, I. C. Kazan, L. Kannan, R. P. Kendle, T. Jamal, M. Barcus, A. Bolia, S. Brimijoin, C. G. Zhan, S. B. Ozkan, T. S. Mor, Plant-expressed cocaine hydrolase variants of butyrylcholinesterase exhibit altered allosteric effects of cholinesterase activity and increased inhibitor sensitivity. *Sci. Rep.* **7**, 10419 (2017).

71. A. Kumar, T. J. Glembo, S. B. Ozkan, The role of conformational dynamics and allostery in the disease development of human ferritin. *Biophys. J.* **109**, 1273–1281 (2015).

72. J. Liu, R. Nussinov, Allostery: An overview of its history, concepts, methods, and applications. *PLOS Comput. Biol.* **12**, e1004966 (2016).

73. T. Zou, B. W. Woodrum, N. Halloran, P. Campitelli, A. A. Bobkov, G. Ghirlanda, S. B. Ozkan, Local interactions that contribute minimal frustration determine foldability. *J. Phys. Chem. B* **125**, 2617–2626 (2021).

74. B. Kolbaba-kartchner, I. Can Kazan, J. H. Mills, S. Banu Ozkan, The role of rigid residues in modulating TEM-1 β-lactamase function and thermostability. *Int. J. Mol. Sci.* **22**, 1–19 (2021).

75. T. Zou, V. A. Risso, J. A. Gavira, J. M. Sanchez-Ruiz, S. B. Ozkan, Evolution of conformational dynamics determines the conversion of a promiscuous generalist into a specialist enzyme. *Mol. Biol. Evol.* **32**, 132–143 (2015).

76. T. Modi, S. Banu Ozkan, Mutations utilize dynamic allostery to confer resistance in TEM-1 β-lactamase. *Int. J. Mol. Sci.* **19**, 1–14 (2018).

77. N. Raddatz, J. P. Castillo, C. Gonzalez, O. Alvarez, R. Latorre, Temperature and voltage coupling to channel opening in transient receptor potential melastatin 8 (TRPM8). *J. Biol. Chem.* **289**, 35438–35454 (2014).

78. H. Kim, T. Zou, C. Modi, K. Dörner, T. J. Grunkemeyer, L. Chen, R. Fromme, M. V. Matz, S. B. Ozkan, R. M. Wachter, A hinge migration mechanism unlocks the evolution of green-to-red photoconversion in GFP-like proteins. *Structure* **23**, 34–43 (2015).

79. B. M. Butler, Z. N. Gerek, S. Kumar, S. B. Ozkan, Conformational dynamics of nonsynonymous variants at protein interfaces reveals disease association. *Proteins* **83**, 428–435 (2015).

80. I. C. Kazan, P. Sharma, M. I. Rahman, A. Bobkov, R. Fromme, G. Ghirlanda, S. B. Ozkan, Design of novel cyanovirin-N variants by modulation of binding dynamics through distal mutations. *eLife* **11**, e67474 (2022).

81. N. J. Ose, B. M. Butler, A. Kumar, I. C. Kazan, M. Sanderford, S. Kumar, S. B. Ozkan, Dynamic coupling of residues within proteins as a mechanistic foundation of many enigmatic pathogenic missense variants. *PLOS Comput. Biol.* **18**, 1–22 (2022).

82. P. Campitelli, T. Modi, S. Kumar, S. Banu Ozkan, The role of conformational dynamics and allostery in modulating protein evolution. *Annu. Rev. Biophys.* **49**, 267–288 (2020).

83. Z. Nevin Gerek, S. Kumar, S. Banu Ozkan, Structural dynamics flexibility informs function and evolution at a proteome scale. *Evol. Appl.* **6**, 423–433 (2013).

84. A. Kumar, B. M. Butler, S. Kumar, S. B. Ozkan, Integration of structural dynamics and molecular evolution via protein interaction networks: A new era in genomic medicine. *Curr. Opin. Struct. Biol.* **35**, 135–142 (2015).

85. M. Ikeguchi, J. Ueno, M. Sato, A. Kidera, Protein structural change upon ligand binding: Linear response theory. *Phys. Rev. Lett.* **94**, 1–4 (2005).

86. R. K. Majhi, S. Saha, A. Kumar, A. Ghosh, N. Swain, L. Goswami, P. Mohapatra, A. Maity, V. Kumar Sahoo, A. Kumar, C. Goswami, Expression of temperature-sensitive ion channel TRPM8 in sperm cells correlates with vertebrate evolution. *PeerJ* **3**, e1310 (2015).

87. X. Lu, Z. Yao, Y. Wang, C. Yin, J. Li, L. Chai, W. Dong, L. Yuan, R. Lai, S. Yang, The acquisition of cold sensitivity during TRPM8 ion channel evolution. *Proc. Natl. Acad. Sci. U.S.A.* **119**, e2201349119 (2022).

88. X. Lu, J. Wu, L. Shi, Y. Zhou, Y. Su, Y. Wang, S. Yang, The adaptive evolution of cold-activated TRPM8 in wildlife vertebrates. *Wildlife Lett.* **1**, 23–31 (2023).

89. H. Matsuura, T. Sokabe, K. Kohno, M. Tominaga, T. Kadowaki, Evolutionary conservation and changes in insect TRP channels. *BMC Evol. Biol.* **9**, 228 (2009).

90. H. N. Turner, K. Armengol, A. A. Patel, N. J. Himmel, L. Sullivan, S. C. Iyer, S. Bhattacharya, E. P. R. Iyer, C. Landry, M. J. Galko, D. N. Cox, The trp channels Pkd2, NompC, and Trpm act in cold-sensing neurons to mediate unique aversive behaviors to noxious cold in drosophila. *Curr. Biol.* **26**, 3116–3128 (2016).

91. N. J. Himmel, J. M. Letcher, A. Sakurai, T. R. Gray, M. N. Benson, D. N. Cox, Drosophila menthol sensitivity and the Precambrian origins of transient receptor potential-dependent chemosensation. *Philos. Trans. R Soc. Lond. B Biol. Sci.* **374**, 20190369 (2019).

92. I. Karpouhtsis, E. Pardali, E. Feggou, S. Kokkini, Z. G. Scouras, P. Mavragani-Tsipidou, Insecticidal and genotoxic activities of oregano essential oils. *J. Agric. Food Chem.* **46**, 1111–1115 (1998).

93. J. S. Dambolena, A. G. López, M. C. Cánepa, M. G. Theumer, J. A. Zygadlo, H. R. Rubinstein, Inhibitory effect of cyclic terpenes (limonene, menthol, menthone and thymol) on Fusarium verticillioides MRC 826 growth and fumonisin B1 biosynthesis. *Toxicon* **51**, 37–44 (2008).

94. A. E. Edris, E. S. Farrag, Antifungal activity of peppermint and sweet basil essential oils and their major aroma constituents on some plant pathogenic fungi from the vapor phase. *Nahrung* **47**, 117–121 (2003).

95. S. Pattnaik, V. R. Subramanyam, M. Bapaji, C. R. Kole, Antibacterial and antifungal activity of aromatic constituents of essential oils. *Microbios* **89**, 39–46 (1997).

96. G. K. A. Hochberg, J. W. Thornton, Reconstructing ancient proteins to understand the causes of structure and function. *Annu. Rev. Biophys.* **46**, 247–269 (2017).

97. M. A. Siddiq, G. K. Hochberg, J. W. Thornton, Evolution of protein specificity: Insights from ancestral protein reconstruction. *Curr. Opin. Struct. Biol.* **47**, 113–122 (2017).

98. J. W. Thornton, E. Need, D. Crews, Resurrecting the ancestral steroid receptor: Ancient origin of estrogen signaling. *Science* **301**, 1714–1717 (2003).

99. G. C. Finnigan, V. Hanson-Smith, T. H. Stevens, J. W. Thornton, Evolution of increased complexity in a molecular machine. *Nature* **481**, 360–364 (2012).

100. C. Wilson, D. Kern, Response to Comment on "Ancient origins of allosteric activation in a Ser-Thr kinase". *Science* **370**, eabd0364 (2020).

101. S. Blanquart, A.-S. Borowiec, P. Delcourt, M. Figeac, C. A. Emerling, A. S. Meseguer, M. Roudbaraki, N. Prevarskaya, G. Bidaux, Evolution of the human cold/menthol receptor, TRPM8. *Mol. Phylogenet. Evol.* **136**, 104–118 (2019).

102. M. D. Andrews, K. Af Forselles, K. Beaumont, S. R. Galan, P. A. Glossop, M. Grenie, A. Jessiman, A. S. Kenyon, G. Lunn, G. Maw, R. M. Owen, D. C. Pryde, D. Roberts, T. D. Tran, Discovery of a selective TRPM8 antagonist with clinical efficacy in cold-related pain. *ACS Med. Chem. Lett.* **6**, 419–424 (2015).

103. S. F. Altschul, T. L. Madden, A. A. Schäffer, J. Zhang, Z. Zhang, W. Miller, D. J. Lipman, Gapped BLAST and PSI-BLAST: A new generation of protein database search programs. *Nucleic Acids Res.* **225**, 3389–3402 (1997).

104. S. F. Altschul, J. C. Wootton, E Michael Gertz, R. Agarwala, A. Morgulis, A. A. Schäffer, Y.-K. Yu, Protein database searches using compositionally adjusted substitution matrices. *FEBS J.* **272**, 5101–5109 (2005).

105. R. C. Edgar, MUSCLE: A multiple sequence alignment method with reduced time and space complexity. *BMC Bioinformatics* **5**, 113 (2004).

106. M. S. Rosenberg, S. Kumar, Traditional phylogenetic reconstruction methods reconstruct shallow and deep evolutionary relationships equally well. *Mol. Biol. Evol.* **18**, 1823–1827 (2001).

107. S. Kumar, G. Stecher, M. Li, C. Knyaz, K. Tamura, MEGA X: Molecular evolutionary genetics analysis across computing platforms. *Mol. Biol. Evol.* **35**, 1547–1549 (2018).

108. S. Kumar, M. Suleski, J. M. Craig, A. E. Kasprowicz, M. Sanderford, M. Li, G. Stecher, S. B. Hedges, TimeTree 5: An expanded resource for species divergence times. *Mol. Biol. Evol.* **39**, msac174 (2022).

109. V. B. Journigan, Z. Feng, S. Rahman, Y. Wang, A. Amin, C. E. Heffner, N. Bachtel, S. Wang, S. Gonzalez-Rodriguez, A. Fernández-Carvajal, G. Fernández-Ballester, J. K. Hilton, W. D. Van Horn, A. Ferrer-Montiel, X. Q. Xie, T. Rahman, Structure-based design of novel biphenyl amide antagonists of human transient receptor potential cation channel subfamily m member 8 channels with potential implications in the treatment of sensory neuropathies. *ACS Chem. Nerosci.* **11**, 268–290 (2020).

110. R. Henson, Flow Cytometry Data Reader and Visualizations, MATLAB Central File Exchange (2023); www.mathworks.com/matlabcentral/fileexchange/8430-flow-cytometry-data-reader-and-visualization.

111. M. K. Khoury, I. Parker, D. W. Aswad, Acquisition of chemiluminescent signals from immunoblots with a digital single-lens reflex camera. *Anal. Biochem.* **397**, 129–131 (2010).

112. K. A. Matreyek, J. J. Stephany, M. A. Chiasson, N. Hasle, D. M. Fowler, An improved platform for functional assessment of large protein libraries in mammalian cells. *Nucleic Acids Res.* **48**, 1–12 (2020).

113. The PyMOL Molecular Graphics System, version 2.4.0, Schrödinger LLC (2020).

114. J. Jumper, R. Evans, A. Pritzel, T. Green, M. Figurnov, O. Ronneberger, K. Tunyasuvunakool, R. Bates, A. Žídek, A. Potapenko, A. Bridgland, C. Meyer, S. A. A. Kohl, A. J. Ballard, A. Cowie, B. Romera-Paredes, S. Nikolov, R. Jain, J. Adler, T. Back, S. Petersen, D. Reiman, E. Clancy, M. Zielinski, M. Steinegger, M. Pacholska, T. Berghammer, S. Bodenstein, D. Silver, O. Vinyals, A. W. Senior, K. Kavukcuoglu, P. Kohli, D. Hassabis, Highly accurate protein structure prediction with AlphaFold. *Nature* **596**, 583–589 (2021).

115. J. Huang, S. Rauscher, G. Nawrocki, T. Ran, M. Feig, B. L. de Groot, H. Grubmüller, A. D. MacKerell Jr., CHARMM36m: An improved force field for folded and intrinsically disordered proteins. *Nat. Methods* **14**, 71–73 (2017).

116. E. L. Wu, X. Cheng, S. Jo, H. Rui, K. C. Song, E. M. Dávila-Contreras, Y. Qi, J. Lee, V. Monje-Galvan, R. M. Venable, J. B. Klauda, W. Im, CHARMM-GUI membrane builder toward realistic biological membrane simulations. *J. Comput. Chem.* **35**, 1997–2004 (2014).

117. S. Jo, T. Kim, V. G. Iyer, W. Im, CHARMM-GUI: A web-based graphical user interface for CHARMM. *J. Comput. Chem.* **29**, 1859–1865 (2008).

118. D. A. Pearlman, D. A. Case, J. W. Caldwell, W. S. Ross, T. E. Cheatham, S. E. DeBolt, D. M. Ferguson, G. Seibel, P. A. Kollman, AMBER, a package of computer programs for applying molecular mechanics, normal mode analysis, molecular dynamics and free energy calculations to simulate the structural and energetic properties of molecules. *Comput. Phys. Commun.* **91**, 1–41 (1995).

119. L. Sawle, K. Ghosh, Convergence of molecular dynamics simulation of protein native states: Feasibility vs self-consistency dilemma. *J. Chem. Theory Comput.* **12**, 861–869 (2016).

120. I. C. Kazan, J. H. Mills, S. B. Ozkan, Allosteric regulatory control in dihydrofolate reductase is revealed by dynamic asymmetry. *Protein Sci.* **32**, e4700 (2023).

121. B. M. Butler, I. C. Kazan, A. Kumar, S. B. Ozkan, Coevolving residues inform protein dynamics profiles and disease susceptibility of nSNVs. *PLOS Comput. Biol.* **14**, e1006626 (2018).

122. C. Atilgan, A. R. Atilgan, Perturbation-response scanning reveals ligand entry-exit mechanisms of ferric binding protein. *PLOS Comput. Biol.* **5**, e1000544 (2009).

123. A. R. Atilgan, S. R. Durell, R. L. Jernigan, M. C. Demirel, O. Keskin, I. Bahar, Anisotropy of fluctuation dynamics of proteins with an elastic network model. *Biophys. J.* **80**, 505–515 (2001).

124. A. O. Stevens, I. C. Kazan, B. Ozkan, Y. He, Investigating the allosteric response of the PICK1 PDZ domain to different ligands with all-atom simulations. *Protein Sci.* **31**, e4474 (2022).

125. F. Sievers, A. Wilm, D. Dineen, T. J. Gibson, K. Karplus, W. Li, R. Lopez, H. McWilliam, M. Remmert, J. Söding, J. D. Thompson, D. G. Higgins, Fast, scalable generation of high-quality protein multiple sequence alignments using Clustal Omega. *Mol. Syst. Biol.* **7**, 539 (2011).

126. C. S. Bond, A. W. Schüttelkopf, ALINE: A WYSIWYG protein-sequence alignment editor for publication-quality alignments. *Acta Crystallogr. D Biol. Crystallogr.* **65**, 510–512 (2009).

127. W. C. Wimley, T. P. Creamer, S. H. White, Solvation energies of amino acid side chains and backbone in a family of host–guest pentapeptides. *Biochem.* **35**, 5109–5124 (1996).
